# ‘Click Chemistry’ Synthesis of Novel Natural Product-Like Caged Xanthones Bearing a 1,2,3-Triazole Moiety with Improved Druglike Properties as Orally Active Antitumor Agents

**DOI:** 10.3390/molecules22111834

**Published:** 2017-10-27

**Authors:** Xiang Li, Yue Wu, Yanyan Wang, Qidong You, Xiaojin Zhang

**Affiliations:** 1 Jiangsu Key Laboratory of Drug Design and Optimization, State Key Laboratory of Natural Medicines, China Pharmaceutical University, Nanjing 210009, China; lixiang@cpu.edu.cn (X.L.); wuyue_1994@163.com (Y.W.); emmawang_88@163.com (Y.W.); 2 Department of Pharmaceutical Engineering, China Pharmaceutical University, Nanjing 211198, China; 3 Department of Medicinal Chemistry, China Pharmaceutical University, Nanjing 21009, China; 4 Department of Organic Chemistry, China Pharmaceutical University, Nanjing 211198, China

**Keywords:** caged xanthones, click chemistry, druglike, antitumor, natural-product-like

## Abstract

**DDO-6101**, a natural-product-like caged xanthone discovered previously in our laboratory based on the pharmacophoric scaffold of the *Garcinia* natural product gambogic acid (GA), shows potent cytotoxicity in vitro, but poor efficacy in vivo due to its poor druglike properties. In order to improve the druglike properties and in vivo antitumor potency, a novel series of ten triazole-bearing caged xanthone derivatives of **DDO-6101** has been efficiently synthesized by ‘click chemistry’ and evaluated for their in vitro antitumor activity and druglike properties. Most of the target compounds have sustained cytotoxicity against A549, HepG2, HCT116, and U2OS cancer cells and possess improved aqueous solubility, as well as permeability. Notably, these caged xanthones are also active towards taxol-resistant or cisplatin-resistant A549 cancer cells. Taking both the in vitro activities and druglike properties into consideration, compound **8g** has been advanced into in vivo efficacy experiments. The results reveal that **8g** (named as **DDO-6318**), both by intravenous or per os administration, are much more potent than the lead **DDO-6101** in A549-transplanted mice models and it could be a promising antitumor candidate for further evaluation.

## 1. Introduction

**Figure 1:**
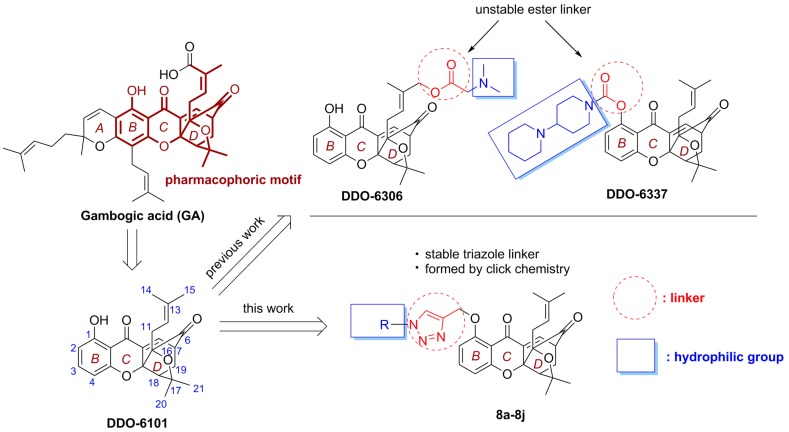
Structures of GA, its simplified caged xanthone analogues and the newly-designed natural product-like compounds.

Natural products (NPs) and natural-product-like compounds inspired by NP structures have played an important role in new drug discovery [1,2]. Notably, in the case of anticancer drugs, more than 70% of the anticancer drugs approved worldwide owe their origins to NPs [1]. Gambogic acid (GA) (Figure 1), a naturally-occurring caged xanthone isolated from *Garcinia* plants, is a promising antitumor agent in clinical study [3,4,5]. Although great efforts has been put into revealing its antitumor mechanisms, the primary direct molecular target of GA is still debatable [6,7,8,9,10,11,12].

Intrigued by the unique structure and therapeutic promise of GA, a large library of its derivatives and simplified caged xanthone analogues has been synthesized and evaluated in our laboratory to explore the structure-activity relationship (SAR) [13,14,15,16], which allows us to understand that the intact BCD ring containing the unique caged scaffold is the minimum pharmacophoric motif essential for its activity. These studies have led to the identification of **DDO-6101** (Figure 1), a natural-product-like caged xanthone with a remarkably simple structure that retains the in vitro antitumor activity of GA [14]. Unfortunately, **DDO-6101** showed poor efficacy in vivo on the tumor growth inhibition in cancer cell-transplanted mice models [13]. This may be due to its poor druglike properties, such as aqueous solubility and cell membrane permeability. Further medicinal chemistry research on **DDO-6101** have revealed that the modifications at the C1 site of the B ring and C15 site in the side chain of the D ring are well-tolerated [17,18], and the introduction of hydrophilic heteroatom-containing groups can improve the druglike properties and enhance the in vivo antitumor potency [13,17,18,19], as shown in the structures of caged xanthone derivatives **DDO-6306** and **DDO-6337** (Figure 1) [17,18]. The main drawbacks of the two types of compounds lie in the unstable ester linker group, which makes the molecules hardly able to survive in the presence of the ester hydrolases in plasma and the acidic environment of the stomach when administered orally.

The copper-catalyzed 1-3-dipolar cycloaddition reaction that couples alkynes and azides forming a 1,2,3-triazole ring, which is commonly known as ‘click chemistry’ due to its ease of use, has become a powerful approach for the rapid construction of pharmacologically-active compounds [20,21]. The 1,2,3-triazole ring is chemically stable against acidic and basic hydrolysis and relatively resistant to metabolic degradation. Moreover, it participates actively in hydrogen bond formation and is beneficial to solubility in water. Herein we present a click chemistry-based linking strategy to couple hydrophilic groups and caged xanthone pharmacophoric motifs to rapidly derive a new series of triazole-bearing caged xanthones (Figure 1) with improved drug-like properties and oral antitumor activity in vivo.

## 2. Results and Discussion

### 2.1. Chemistry

**Scheme 1:**
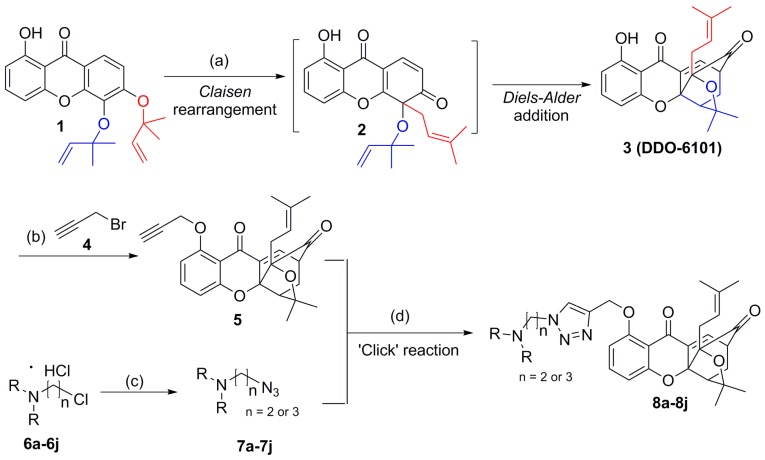
The synthetic route of designed compounds. Reagents and conditions: (**a**) DMF, 120 °C, 2 h, 78.5%; (**b**) **4** (1.1 equiv.), K_2_CO_3_ (1.1 equiv.), DMF, r.t., overnight, 86.7%; (**c**) NaN_3_ (1.5 equiv.), K_2_CO_3_ (3 equiv.), H_2_O, 75 °C, 15 h; (**d**) **7a–7j** (2 equiv. relative to **5**), CuSO_4_·5H_2_O (0.01 equiv.), sodium ascorbate (0.3 equiv.), *t*-BuOH/H_2_O (*v*:*v* = 1:1), r.t., overnight, 90.2–96.4%. For the detailed substitution groups for **8a**–**8j** see Table 1.

The designed caged xanthane derivatives **8a**–**8j** and their synthetic routes are shown in Scheme 1. The starting material **1** was prepared according to our previously-reported procedure [14]. Heating **1** in DMF gave rise to the caged xanthone **3** (**DDO-6101**) via a Claisen rearrangement and Diels-Alder addition cascade. The hydroxyl group at the C1 position is considered as a potential modification site. Treatment of **3** with 3-bromoprop-1-yne (**4**) in the presence of potassium carbonate produced the alkyne intermediate **5**. Meanwhile, the substitution reaction between **6a**–**6j** and sodium azide provided the azide intermediates **7a**–**7j** quantitatively, which were directly used for the following reaction without further purification. The click reaction between alkyne **5** and azide **7a**–**7j** in the presence of CuSO_4_·5H_2_O and sodium ascorbate led to the target triazole-bearing caged xanthone derivatives **8a**–**8j** in excellent yields. The chemical structures of these derivatives were characterized using ^1^H-nuclear magnetic resonance (NMR), infrared (IR) spectroscopy, and high-resolution mass spectrometry (HRMS). Representative compounds **8a**, **8c**, **8e**, **8g** and **8h** were further characterized by ^13^C-NMR. All of the target compounds yielded acceptable purity (>95%) by high-performance liquid chromatography (HPLC) analysis. ^1^H-NMR, ^13^C-NMR, HRMS spectra for the target compounds are shown in supplementary materials 1.

### 2.2. In Vitro Cytotoxic Effects

The antiproliferative activity of the ten synthesized caged xanthone derivatives, as well as the lead compound **DDO-6101** and GA were assessed with the 3-(4,5-dimethylthiazol-2-yl)-2,5-diphenyltetrazolium bromide (MTT) assay, as previously reported [18], against six human cancer cell lines, including the human lung carcinoma A549 cell line, taxol-resistant A549 cell line (A549/taxol), cisplatin-resistant A549 cell line (A549/cisplatin), the human hepatocellular carcinoma HepG2 cell line, the human colon carcinoma HCT-116 cell line, and the human glioblastoma U251 cell line. The antiproliferative activities, expressed as *IC*_50_ values, are summarized in Table 1. **DDO-6101** and GA were used as positive controls for the in vitro assay.

As shown in Table 1, in general, most of the designed triazole-bearing caged xanthones **8a**–**8h** inhibited potent antiproliferative activities against A549, HepG2, HCT116, and U2OS cells, with *IC*_50_ values in the low micromolar range, which are comparable to those of **DDO-6101** and natural product GA. Interestingly, it was observed that these compounds showed comparable, or even better, cytotoxic activity against A549/taxol and A549/cisplatin cell lines when compared to A549 cells, as well as three other cancer cell lines. The results indicated that these xanthone derivatives with triazole- and nitrogen-containing side chains at the C1 site function in cancer cells and multidrug-resistant ones, as well.

Different compounds showed different activities against the six cancer cell lines. **8f**, **8g**, **8h** and **8j** showed potent cytotoxicity against A549 cells, with growth inhibition *IC*_50_ values of 1.07 ± 0.28, 0.31 ± 0.02, 1.03 ± 0.06, and 0.93 ± 0.08 μM, respectively, which were much more active than both the **DDO-6101** and GA. Moreover, the taxol-resistant and cisplatin-resistant A549 cell lines were both significantly sensitive to **8g** and **8h**, with *IC*_50_ values ranging from 0.33 to 0.55 μM. The HepG2 cell line was sensitive to **8b** and **8c**, with *IC*_50_ values of 1.36 ± 0.16 and 1.19 ± 0.31 μM, respectively, which were more potent than **DDO-6101** and GA, while in the HCT116 cell lines **8g** exerted the best cytotoxic activity with an *IC*_50_ value of 0.28 ± 0.03 μM. Although many other compounds, such as **8c**, **8d**, **8h** and **8j**, possessed low micromolar activities against HCT116 cells, they were less active than **DDO-6101**. In the case of the U2OS cell line, it was generally less sensitive to this series of compounds as compared to the five other cell lines, with *IC*_50_ values ranging from 0.73 to 9.51 μM. However, eight compounds **8a**–**8h** showed more potent cytotoxicity than **DDO-6101** against U2OS, among which **8h** was the most active with an *IC*_50_ value of 0.73 ± 0.07 μM.

According to the in vitro data in Table 1, it was found that the introduction of the triazole moiety with nitrogen-containing hydrophilic groups, especially the morpholino and 4-methylpiperizin-1-yl groups as in **8g**–**8j**, could improve the cytotoxic activity against most of the tested cancer cell lines. Few differences in cytotoxicity were found between compounds with different carbon chain lengths (*n* = 2 or 3). Among this series of target compounds, **8g** showed the most potent inhibitory activities in vitro towards A549, A549/taxol, A549/cisplatin, and HCT116 cells, with *IC*_50_ values of 0.31 ± 0.02, 0.42 ± 0.05, 0.33 ± 0.07, and 0.28 ± 0.03, respectively, which was more active in vitro than the lead **DDO-6101**. It was noteworthy that **8g** displayed approximately eight-fold higher activity than **DDO-6101** in A549 cells.

### 2.3. Drug-Like Property Evaluation

**Figure 2:**
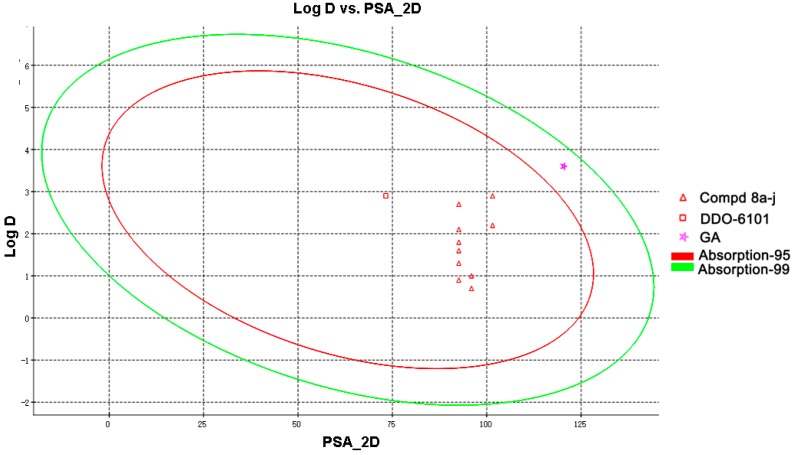
Plot of log P versus PSA_2D.

In parallel with the in vitro antitumor activity testing, the physicochemical properties of the target caged xanthone derivatives were theoretically predicted and experimentally determined in an effort to identify potential druglike compounds prior to the time-consuming and costly development and optimization of derivatives that may ultimately fail in efficacy experiments in vivo. The partition coefficient (logD) was experimentally determined on a Gemini Profiler instrument (pION, Inc., Woburn, MA, USA) by a potentiometric titration method [13]. The polar surface area (PSA), as well as intestinal absorption level, were theoretically predicted by Accelrys Discovery Studio 4.0 (BIOVIA, San Diego, CA, USA) using the ADMET protocol [17]. The aqueous solubility were experimentally measured using HPLC at the maximum absorption wavelength of 290 nm based on the results of full-wave scanning [18]. The permeability coefficient (Pe) was determined using a standard parallel artificial membrane permeability assay (PAMPA) on a PAMPA Explorer instrument (pION) [17]. Ketoprofen and propranolol were used as internal standards for permeability. These data are shown in Table 2 and Figure 2.

Whether a drug can be well-absorbed intestinally after oral administration depends on the intrinsic properties of the molecule. The polar surface area and distribution coefficient are the two main aspects. Analysis of a plot of the logD versus PSA_2D of the caged xanthones **8a**–**8j** (Table 2 and Figure 2) showed that all of the compounds fell within the ellipse region of 95% (aborption_level = 0) while GA fell outside the ellipse region of 95% (aborption_level = 1). These results suggested that the xanthone derivatives are likely to have favorable intestinal absorption. 

Meanwhile, compounds **8a**–**8j** were set to determine experimentally their aqueous solubility and permeability, which are the two important properties reflecting the ability of molecules to penetrate to the therapeutic target across the cell membrane and watery environment in vivo. **DDO-6101** and GA were used as reference compounds for comparison. As shown in Table 2, all the xanthone derivatives displayed aqueous solubility that was significantly improved over that of **DDO-6101** and GA. This may be attributed to the introduction of 1,2,3-triazole and hydrophilic groups, as well as the disruption of intermolecular hydrogen bonds between C1-OH and the adjacent carbonyl group in the structure of **DDO-6101**. Surprisingly, compounds with a chain of three carbon atom (e.g., **8b**, **8d**, **8f**, **8h**, **8j**) showed slightly more solubility than the corresponding ones with a chain of two carbon atoms (e.g., **8a**, **8c**, **8e**, **8g**, **8i**). Moreover, it was found that compounds **8g**–**8j** were more soluble than that of **8a**–**8f**. The results suggested that the introduction of hydrophilic groups, such as morpholino and 4-methylpiperazin-1-yl groups, resulted in a significant increase in solubility.

**Figure 3:**
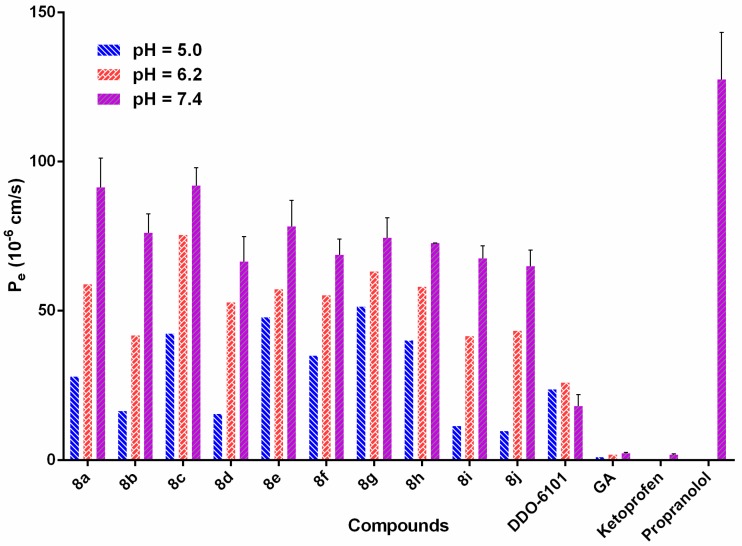
Experimental determination of membrane permeability of compounds **8a**–**8j** at different pH values.

In addition, the introduction of triazole moiety and hydrophilic groups to the C1 site of **DDO-6101** also led to an improvement in permeability. As shown in Figure 3 (for the detailed data see Supplementary Materials Table S1), most of the xanthone derivatives displayed improved permeability than **DDO-6101** (P_e_ = 18.0–23.6 × 10^–6^ cm/s) and GA (P_e_ = 0.9–2.3 × 10^–6^ cm/s). The permeability decreased as the aqueous environment turned to be more acidic. Among the compounds tested, **8e** and **8g** showed preferable permeability under different pH from 5.0 to 7.4 with P_e_ values of 47.0–78.2 × 10^–6^ cm/s and 51.3–74.4 × 10^–6^ cm/s, respectively. This suggested that these xanthone derivatives are likely to have favorable properties of absorption and distribution in vivo.

### 2.4. In Vivo Antitumor Evaluations of Compound ***8h***

In the light of the in vitro antitumor activity and druglike properties, such as aqueous solubility and permeability, we selected compound **8h** to determine its in vivo efficacy. The lung cancer A549 transplanted mouse model was employed. **DDO-6101** and antitumor drug 5-fluorouracil (5-FU) were also tested for comparison. As shown in Table 3, the tumor growth inhibitory rates of **DDO-6101** and 5-FU were 34.56% and 64.71%, respectively, at 20 mg/kg twice daily doses, over a period of two weeks. **8g** displayed significant inhibitory effect on the growth of inoculated A549 in mice in a dose-dependent manner, with 30.88%, 52.21% and 71.32% inhibition in tumor growth, at 5, 10 and 20 mg/kg twice daily doses, respectively. No vascular irritation or weight loss was observed in any of these in vivo tests. Compared with **DDO-6101**, the triazole-bearing derivative **8g** was much more potent in vivo on the inhibition of the growth of inoculated A549 in mice. This may be attributed to the increased in vitro cytotoxicity and improved druglike properties, such as aqueous solubility and cell membrane permeability of **8g** compared to **DDO-6101**.

Subsequently, **8g** was set to determine further its in vivo efficacy with per os administration on the growth of inoculated A549 in mice using **DDO-6101** as a comparison control. As shown in Table 4, **8g** displayed much more inhibitory potency on the growth of inoculated A549 in mice than **DDO-6101**. According to the data, 66.43% inhibition in tumor growth for **8g** was observed at 50 mg/kg daily oral dose, over a period of one week, while the inhibitory rate of **DDO-6101** was 21.43%. Thus, compound **8g** was identified as a new and orally-active natural-product-like caged xanthone and also a promising antitumor agent for further clinical development.

## 3. Materials and Methods

### 3.1. General Information

All reagents were purchased from commercial sources and, unless otherwise noted, were used without further purification. Organic solutions were concentrated in a rotary evaporator (Büchi Rotavapor, Büchi, Uster, Switzerland) below 45 °C (70 °C for DMF) under reduced pressure. Reactions were monitored by thin-layer chromatography (TLC, Qingdao Haiyang Chemical Co., Ltd., Qingdao, China) on 0.25 mm silica gel plates (GF254) and visualized under UV light. Silica gel (60 Å, 300–400 mesh) was used for flash column chromatography. Melting points were determined with a Melt-Temp II apparatus (Barnstead/Thermolyne Corp., Dubuque, IA, USA) and were reported without correction. IR spectra were recorded on a Nicolet iS10 Avatar FT-IR spectrometer (Thermo Fisher Scientific, Waltham, MA, USA) using KBr film. The ^1^H-NMR and ^13^C-NMR spectra were measured on a Bruker AV-300 instrument (Bruker, Billerica, MA, USA) using deuterated solvents with tetramethylsilane (TMS) as an internal standard. High-resolution mass spectra (HRMS) were recorded on a Water Q-Tof micro mass spectrometer (Waters, Milford, MA, USA). The purity is verified by the HPLC study performed on an Amethyst C18-P (4.6 × 150 mm, 5 μm, Merck, Darmstadt, Germany) column using a mixture of solvent methanol or acetonitrile/water at the flow rate of 2 mL/min and peak detection at 240 nm under UV.

Cancer cell lines were obtained from Cell Bank of Shanghai, Chinese Academy of Sciences. Cells were cultured in 90% RPMI 1640 medium (GIBCO, Invitrogen Corporation, New York, NY, USA) supplemented with 10% fetal bovine serum (Sijiqing Biotechnology Co., Hangzhou, China), 100 U/mL benzyl penicillin, and 100 μg/mL streptomycin in a humidified environment with 5% CO_2_ at 37 °C. All the tested compounds were dissolved in DMSO at a concentration of 0.01 mol/L and stored at −4 °C.

### 3.2. Synthesis of Compound ***3***

Compound **1**. (5.0 g, 13.16 mmol) was dissolved in DMF (20 mL) and the reaction solution was stirred at 120 °C under nitrogen for 2 h. The reaction mixture was then cooled to room temperature and concentrated. The residue was purified by column chromatography (16:1 petroleum ether/ethyl acetate) to afford compound **3** (**DDO-6101**) (3.925 g, 78.5%) as a yellow solid. m.p.: 130–132 °C. ^1^H-NMR (300 MHz, CDCl_3_): 0.95 (s, 3H), 1.18–1.25 (m, 4H), 1.30 (s, 3H), 1.61 (s, 3H), 2.26 (dd, *J*_1_ = 13.5 Hz, *J*_2_ = 4.5 Hz, 1H), 2.37 (d, *J* = 9.6 Hz, 1H), 2.54 (d, *J* = 7.8 Hz, 2H), 3.44 (dd, *J*_1_ = 6.9 Hz, *J*_2_ = 4.5 Hz, 1H), 4.32–4.36 (m, 1H), 6.43 (dd, *J*_1_ = 8.1 Hz, *J*_2_ = 0.9 Hz, 1H), 6.45 (dd, *J*_1_ = 8.1 Hz, *J*_2_ = 0.9 Hz, 1H), 7.32 (t, *J* = 8.1 Hz, 1H), 7.41 (d, *J* = 9.6 Hz, 1H), 12.00 (s, 1H); HRMS (ESI): calcd. for C_23_H_24_O_5_ [M + H]^+^ 381.1697, found 381.1715; HPLC 99.5% (80% acetonitrile in water, tR: = 4.7 min).

### 3.3. Synthesis of Compound ***5***

To a stirring solution of compound **3** (3.8 g, 0.1 mol) in DMF (50 mL) was added K_2_CO_3_ (15.18 g, 0.11 mol) and 3-bromoprop-1-yne (12.83 mL, 0.11 mol). The reaction mixture was stirred at room temperature overnight. A saturated ammonium chloride aqueous (200 mL) was added. The mixture was extracted by ethyl acetate (100 mL × 3). The ethyl acetate layer was partitioned, combined, washed with brine (50 mL × 3), dried over magnesium sulfate, and concentrated. The residue was purified by column chromatography (5:1 petroleum ether/ethyl acetate) to afford compound **5** (3.625 g, 86.7%) as a white solid. m.p. 151–153 °C; ^1^H-NMR (300 MHz, CDCl3): δ 1.11 (s, 3H), 1.27–1.31 (m, 4H), 1.42 (s, 3H), 1.72 (s, 3H), 2.33 (dd, *J*_1_ = 8.8 Hz, *J*_2_ = 4.5 Hz, 1H), 2.42 (d, *J* = 9.6 Hz, 1H), 2.54 (t, *J* = 2.3 Hz,1H), 2.63 (d, *J* = 7.7 Hz, 2H), 3.44–3.48 (m, 1H), 4.51 (t, *J* = 7.8 Hz, 1H), 4.86 (d, *J* = 2.3 Hz, 2H), 6.73–6.76 (m, 2H), 7.32 (d, *J* = 6.9 Hz, 1H), 7.41–7.47 (t, *J* = 8.4 Hz, 1H); IR (KBr, cm^−1^): 3253, 2993, 1738, 1672, 1613, 1600, 1573, 1467, 1109, 1061, 848, 799, 697; HRMS (ESI): calcd. for C_26_H_27_O_5_ [M + H]^+^ 419.1853, found 419.1864. HPLC purity: 97.7% (elution: 70% methanol in water, tR: 12.4 min).

### 3.4. Synthesis of Compounds ***8a**–**8j***

To a solution of corresponding reactant **6a**–**6j** (4 mmol) in water (30 mL) was added K_2_CO_3_ (1.656 g, 12 mmol) and sodium azide (0.390 g, 6 mmol). The reaction mixture was stirred at 75 °C for 10 h, then cooled to room temperature and extracted by dichloromethane (15 mL × 3). The organic layer was partitioned, combined, washed with bine (15 mL), dried over magnesium sulfate and concentrated to afford crude product **7a**–**7j**. The crude residue **7a**–**7j** was then dissolved in a mixture solvent of *t*-BuOH/H_2_O (*v*:*v* = 1:1, 15 mL). Compound **5** (0.836 g, 2 mmol), sodium ascorbate (119 mg, 0.6 mmol), and CuSO_4_·5H_2_O (5 mg, 0.02 mmol) were added into the mixture. The reaction mixture was stirring at room temperature under N_2_ protection overnight. Then water (200 mL) was added and the mixture was extracted by dichloromethane (15 mL × 3). The organic layer was partitioned, combined, washed with bine (15 mL), dried over magnesium sulfate and concentrated. The residue was purified by column chromatography (20:1 dichloromethane/methanol) to afford the products **8a**–**8j**.

Compound **8a**. Yield 91.5%. Slight yellow solid. m.p. 156–158 °C; ^1^H-NMR (300 MHz, CDCl_3_): δ 1.01 (s, 3H), 1.18–1.24 (m, 4H), 1.31 (s, 3H), 1.63 (s, 3H), 2.22–2.27 (m, 7H), 2.33 (d, *J* = 9.5 Hz, 1H), 2.54 (d, *J* = 7.8 Hz, 2H), 2.73 (t, *J* = 6.5 Hz, 2H), 4.37–4.46 (m, 1H), 4.31–4.42 (m, 3H), 5.26 (s, 2H), 6.61–6.69 (m, 2H), 7.20–7.23 (m, 1H), 7.35 (t, *J* = 8.3 Hz, 1H), 7.95 (s, 1H); ^13^C-NMR (75 MHz, CDCl_3_): δ 16.48, 25.03, 25.13, 28.48, 29.76, 30.38, 44.89, 46.21, 47.84, 47.99, 58.22, 63.35, 82.85, 83.91, 89.41, 105.73, 110.15, 110.55, 118.13, 123.04, 131.86, 134.15, 135.71, 136.02, 143.59, 159.09, 160.66, 175.09, 202.64; IR (KBr, cm^−1^): 3197, 3109, 2996, 1737, 1667, 1603, 1474, 1460, 1401, 1258, 1111, 1066, 846, 791, 669; HRMS (ESI): calcd. for C_30_H_37_N_4_O_5_ [M + H]^+^ 533.2758, found 533.2774; HPLC purity: 100% (elution: 75% methanol in water, tR: 4.63 min).

Compound **8b**. Yeild 92.4%. Slight yellow solid. m.p. 133–136 °C; ^1^H-NMR (300 MHz, CDCl_3_): δ 1.08 (s, 3H, C_14_-H), 1.25–1.29 (m, 4H), 1.37 (s, 3H), 1.69 (s, 3H, C_20_-H), 2.15–2.25 (m, 2H), 2.28–2.41 (m, 8H), 2.48–2.53 (m, 2H), 2.61 (d, *J* = 7.7 Hz, 2H), 3.43–3.47 (m, 1H), 4.45–4.49 (m, 3H), 5.31 (s, 2H), 6.71 (t, *J* = 7.8 Hz, 2H), 7.26–7.29 (m, 1H), 7.43 (t, *J* = 8.3 Hz, 1H), 7.98 (s, 1H); IR (KBr, cm^−1^): 3196, 3106, 2996, 1737, 1669, 1603, 1475, 1401, 1258, 1111, 1066, 846, 791, 669; HRMS (ESI): calcd. for C_31_H_39_N_4_O_5_ [M + H]^+^ 547.2915, found 547.2916; HPLC purity: 100% (elution: 75% methanol in water, tR: 4.47 min).

Compound **8c**. Yeild 90.2%. Slight yellow solid. m.p. 168–170 °C; ^1^H-NMR (300 MHz, CDCl_3_): δ 1.08 (s, 3H), 1.25–1.29 (m, 4H, C_15_-H), 1.37 (s, 3H), 1.70 (s, 3H), 1.78–1.81 (m, 4H), 2.33 (dd, *J*_1_ = 8.8 Hz, *J*_2_ = 4.5 Hz, 1H), 2.42 (d, *J* = 9.6 Hz, 1H), 2.60–2.62 (m, 6H), 3.04 (t, *J* = 6.7 Hz, 2H), 3.44–3.47 (m, 1H), 4.49–4.56 (m, 3H), 5.33 (s, 2H), 6.68–6.76 (m, 2H), 7.27–7.29 (m, 1H), 7.42 (t, *J* = 8.4 Hz, 1H), 8.05 (s, 1H); ^13^C-NMR (75 MHz, CDCl_3_): δ 16.49, 23.02, 25.04, 25.13, 28.48, 28.50, 29.77, 46.22, 48.00, 48.66, 53.49, 54.77, 63.31, 82.85, 83.92, 89.45, 105.70, 110.15, 110.57, 118.12, 123.19, 131.89, 134.17, 135.69, 136.01, 143.65, 159.06, 160.67, 175.07, 202.67; IR (KBr, cm^−1^): 3196, 3110, 2996, 1737, 1668, 1603, 1474, 1458, 1400, 1258, 1110, 1066, 847, 791, 669; HRMS (ESI): calcd. for C_32_H_39_N_4_O_5_ [M + H]^+^ 559.2915, found 559.292; HPLC purity: 95.1% (elution: 75% methanol in water, tR: 4.45 min).

Compound **8d**. Yeild 96.4%. Slight yellow solid. m.p. 144–146 °C; ^1^H-NMR (300 MHz, CDCl_3_): δ 1.10 (s, 3H), 1.26–1.33 (m, 4H), 1.39 (s, 3H), 1.72 (s, 3H), 1.83 (m, 4H), 2.14–2.23 (m, 2H), 2.33 (dd, *J*_1_ = 9.2 Hz, *J*_2_ = 4.3 Hz, 1H), 2.42 (d, *J* = 9.6 Hz, 1H), 2.53–2.64 (m, 8H), 3.45–3.49 (m, 1H), 4.47–4.54 (m, 3H), 5.34 (s, 2H), 6.73 (t, *J*_2_ = 8.7 Hz, 2H), 7.29–7.31 (m, 1H), 7.44 (t, *J* = 8.4 Hz, 1H), 7.98 (s, 1H); IR (KBr, cm^−1^): 3196, 3110, 2996, 1736, 1670, 1604, 1474, 1458, 1401, 1263, 1110, 1066, 847, 791, 669; HRMS (ESI): calcd. for C_33_H_41_N_4_O_5_ [M + H]^+^ 573.3071, found 573.3080; HPLC purity: 98.8% (elution: 75% methanol in water, tR: 4.46 min).

Compound **8e**. Yeild 92.8%. Slight yellow solid. m.p. 173–176 °C; ^1^H-NMR (300 MHz, CDCl_3_): δ 1.07 (s, 3H, C_14_-H), 1.28 (m, 4H), 1.36 (s, 3H), 1.42–1.44 (m, 2H), 1.56–1.60 (m, 4H), 1.69 (s, 3H), 2.31 (dd, *J*_1_ = 9.0 Hz, *J*_2_ = 4.5 Hz, 1H), 2.39 (d, *J* = 9.6 Hz, 1H), 2.45 (m, 4H), 2.60 (d, *J* = 7.7 Hz, 2H), 2.80 (t, *J* = 6.5 Hz, 2H), 3.42–3.46 (m, 1H), 4.46–4.50 (m, 3H), 5.32 (s, 2H), 6.67–6.75 (m, 2H), 7.27 (d, *J* = 6.8 Hz, 1H), 7.41 (t, *J* = 8.3 Hz, 1H), 8.07 (s, 1H); ^13^C-NMR (75 MHz, CDCl_3_): δ 16.45, 23.62, 25.04, 25.14, 25.28, 28.48, 28.50, 29.77, 46.21, 47.31, 47.99, 54.00, 57.66, 63.37, 82.85, 83.93, 89.45, 105.70, 110.16, 110.54, 118.12, 123.21, 131.80, 134.18, 135.67, 136.05, 143.56, 159.08, 160.65, 175.00, 202.69; IR (KBr, cm^−1^): 3200, 3111, 2996, 1738, 1668, 1603, 1476, 1458, 1400, 1259, 1116, 1064, 791, 669; HRMS (ESI): calcd. for C_33_H_41_N_4_O_5_ [M + H]^+^ 573.3071, found 573.3084; HPLC purity: 100% (elution: 75% methanol in water, tR: 4.69 min).

Compound **8f**. Yeild 93.6%. Slight yellow solid. m.p. 125–128 °C; ^1^H-NMR (300 MHz, CDCl_3_): δ 1.07 (s, 3H), 1.23–1.30 (m, 4H), 1.36 (s, 3H, C_19_-H), 1.42–1.44 (m, 2H), 1.57–1.61 (m, 4H), 1.68 (s, 3H), 2.10–2.15 (m, 2H), 2.29–2.40 (m, 8H), 2.60 (d, *J* = 7.7 Hz, 2H), 3.42–3.46 (m, 1H), 4.42–4.49 (m, 3H), 5.31 (s, 2H), 6.67–6.73 (m, 2H), 7.26–7.28 (m, 1H), 7.41 (t, *J* = 8.3 Hz, 1H), 7.93 (s, 1H); IR (KBr, cm^−1^): 3200, 3111, 2996, 1739, 1668, 1605, 1478, 1462, 1401, 1267, 1116, 1064, 878, 791, 669; HRMS (ESI): calcd. for C_34_H_43_N_4_O_5_ [M + H]^+^ 587.3228, found 587.3228; HPLC purity: 98.5% (elution: 75% methanol in water, tR: 4.45 min).

Compound **8g**. Yeild 96.4%. Slight yellow solid. m.p. 175–178 °C; ^1^H-NMR (300 MHz, CDCl_3_): δ 1.09 (s, 3H), 1.26–1.32 (m, 4H), 1.37 (s, 3H, C_19_-H), 1.70 (s, 3H), 2.32 (dd, *J*_1_ = 8.9 Hz, *J*_2_ = 4.5 Hz, 1H), 2.40 (d, *J* = 9.5 Hz, 1H), 2.51 (t, 4H, *J* = 4.5 Hz), 3.72 (d, *J* = 7.6 Hz, 2H), 2.85 (t, *J* = 6.2 Hz, 2H), 3.44–3.48 (m, 1H), 3.72 (t, *J* = 4.7 Hz, 4H), 4.48–4.52 (m, 3H), 5.33 (s, 2H), 6.72 (t, *J* = 7.7 Hz, 2H), 7.27–7.29 (m, 1H), 7.43 (t, *J* = 8.3 Hz, 1H), 8.14 (s, 1H); ^13^C-NMR (75 MHz, CDCl_3_): δ 16.47, 25.03, 25.12, 28.48, 29.77, 30.37, 46.22, 46.93, 48.02, 52.97, 57.29, 63.44, 66.27, 82.85, 83.90, 89.49, 105.50, 110.10, 110.59, 118.15, 123.16, 131.90, 134.11, 135.71, 136.02, 143.79, 159.05, 160.70, 175.03, 202.61; IR (KBr, cm^−1^): 3200, 3111, 2993, 1737, 1668, 1603, 1474, 1457, 1401, 1258, 1109, 1066, 872, 846, 791, 689; HRMS (ESI): calcd. for C_32_H_39_N_4_O_6_ [M + H]^+^ 575.2864, found 575.2892; HPLC purity: 95.8% (elution: 75% methanol in water, tR: 8.56 min).

Compound **8h**. Yeild 91.8%. Slight yellow solid. m.p. 143–145 °C; ^1^H-NMR (300 MHz, CDCl_3_): δ 1.09 (s, 3H), 1.26–1.30 (m, 4H), 1.38 (s, 3H), 1.71 (s, 3H), 2.10–2.17 (m, 2H), 2.30–2.44 (m, 8H), 2.60 (d, *J* = 7.7 Hz, 1H), 3.45–3.48 (m, 1H), 3.72 (t, *J* = 4.7 Hz, 4H), 4.46–4.53 (m, 3H), 5.33 (s, 2H), 6.72 (t, *J* = 7.6 Hz, 2H), 7.27–7.30 (m, 1H), 7.44 (t, *J* = 8.4 Hz, 1H), 7.97 (s, 1H); ^13^C-NMR (75 MHz, CDCl_3_): δ 16.48, 25.06, 25.12, 26.47, 28.49, 28.51, 29.77, 46.20, 47.65, 47.98, 53.00, 54.43, 63.41, 66.41, 82.87, 83.89, 89.44, 105.51, 110.05, 110.62, 118.15, 122.82, 131.88, 134.14, 135.77, 135.99, 143.73, 159.00, 160.68, 175.09, 202.62; IR (KBr, cm^−1^): 3201, 3112, 2963, 1737, 1668, 1604, 1477, 1457, 1400, 1267, 1115, 1064, 878, 844, 793, 696; HRMS (ESI): calcd. for C_33_H_41_N_4_O_6_ [M + H]^+^ 589.3021, found 589.3037; HPLC purity: 95.7% (elution: 75% methanol in water, tR: 4.12 min).

Compound **8i**. Yeild 90.6%. Slight yellow solid. m.p. 171–175 °C; ^1^H-NMR (300 MHz, CDCl_3_): δ 1.07 (s, 3H), 1.25–1.30 (m, 4H), 1.37 (s, 3H), 1.69 (s, 3H), 2.30–2.62 (m, 15H), 2.85 (t, *J* = 6.4 Hz, 2H)_)_, 3.43–3.47 (m, 1H), 4.46–4.50 (m, 3H), 5.32 (s, 2H), 6.68–6.75 (m, 2H), 7.26–7.29 (m, 1H), 7.42 (t, *J* = 8.4 Hz, 1H), 8.05 (s, 1H); IR (KBr, cm^−1^): 3210, 3113, 2967, 1738, 1672, 1602, 1475, 1457, 1400, 1109, 1064, 791, 685; HRMS (ESI): calcd. for C_33_H_42_N_5_O_5_ [M + H]^+^ 588.3180 found 588.3189; HPLC purity: 95.9% (elution: 70% methanol in water, tR: 3.13 min).

Compound **8j**. Yeild 91.2%. Slight yellow solid. m.p. 126–129 °C; ^1^H-NMR (300 MHz, CDCl_3_): δ 1.10 (s, 3H), 1.24–1.33 (m, 4H), 1.39 (s, 3H), 1.71 (s, 3H), 2.05–2.15 (m, 2H), 2.31–2.48 (m, 15H), 2.63 (d, *J* = 7.5 Hz, 1H), 3.45–3.49 (m, 1H), 4.44–4.51 (m, 3H), 5.34 (s, 2H), 6.70–6.76 (m, 2H), 7.28–7.31 (m, 1H), 7.44 (t, *J* = 8.3 Hz, 1H), 7.96 (s, 1H); IR (KBr, cm^−1^): 3103, 2973, 1741, 1683, 1616, 1605, 1478, 1462, 1399, 1262, 1110, 1013, 878, 792, 695; HRMS (ESI): calcd. for C_34_H_44_N_5_O_5_ [M + H]^+^ 602.3337 found 602.3346; HPLC purity: 97.5% (elution: 75% methanol in water, tR: 4.39 min).

### 3.5. Cell Proliferation Assay

The inhibition of tumour cell growth was measured by a modified tetrazolium (MTT) salt assay. Cells were cultured in a 96-well plate and treated with culture medium alone or different concentrations of the compounds. The cells were incubated for 72 h, and then the MTT assay reagent was added. After a 4 h incubation, the formazan product was quantitated at 570 nm. The *IC*_50_ values (the concentrations that gave rise to 50% inhibition of cell viability) were calculated using GraphPad Prism 6 using a variable slope (four parameters).

### 3.6. Determination of the Druglike Properties

A HPLC system with an ultraviolet (UV) detector was used to detect the solubility of the compounds. The optimal wavelength was 290 nm. Ethanol/water (85:15) was an efficient eluent at a flow rate of 0.5 mL/min. The compounds were stirred in deionized water overnight to maximize the retention of the compounds in solution. Then, the solutions were filtered and the supernatant was used as a fluid sample for HPLC detection. The permeability (Pe) of the compounds was tested with the help of a standard PAMPA (pION). PAMPA was performed on a PAMPA Explorer instrument (pION) with the PAMPA Explorer command software (Version 3.7.4.1). The compounds were diluted to 10 mM with the system solution buffer (pH 7.4). Then, 150 μL of the diluted compound solution was transferred to a UV plate, and the UV spectrum was collected and used as a reference. Then, paint the membrane with 5 μL of gastrointestinal tract (GIT) lipid. The acceptor chamber was filled with 200 μL of acceptor solution buffer, and the donor chamber was filled with 200 μL of diluted compound solution. The PAMPA sandwich was assembled and left at 25 °C for 4 h. The UV spectra (240–500 nm) from the donor and the acceptor were collected. The permeability coefficient was calculated with the PAMPA Explorer Command software (Version 3.7.4.1) based on the area under the curve (AUC) of the reference plate, the donor plate, and the acceptor plate. The permeability coefficient of the compound was tested for four times, and the data are shown as the average values. Ketoprofen (1.8 × 10^−6^ cm/s) and propranolol (127.5 × 10^−6^ cm/s) were used as the standards in this assay.

### 3.7. In Vivo Tumor Growth Inhibition Assay

Kunming mice with body weights of 18–22 g were transplanted with lung cancer A549 cells according to protocols of transplant tumor research, as previously reported [22]. Twenty-four hours after tumor transplantation, animals were weighed and randomly divided into five groups. All test drugs were given through injections 24 h after tumor transplantation. The treated groups were intravenously injected or administered per os different doses of test compounds, respectively. The negative control group received 0.9% normal saline. Treatments were conducted at a frequency of one dose per two days for the intravenous group or one dose per day for the per os group for two or one week, respectively. All animals were sacrificed 24 h after the final treatment. All data are presented as mean ± SD and the statistical significance was evaluated by *t*-test.

## 4. Conclusions

In summary, a click chemistry-based optimization of the C1-OH site of the lead compound **DDO-6101**, utilizing a strategy that concerned both the in vitro activity and druglike property, successfully led to the discovery of a novel series of natural-product-like triazole-bearing caged xanthones with improved druglike properties as orally-active antitumor agents in vivo, represented by compound **8g**. It showed potent inhibitory activities in vitro towards A549, A549/taxol, A549/cisplatin, and HCT116 cells, with *IC*_50_ values of 0.31 ± 0.02, 0.42 ± 0.05, 0.33 ± 0.07, and 0.28 ± 0.03, respectively, which was more active than the lead **DDO-6101**. Notably, **8g** displayed 66.43% growth inhibition in the lung cancer cell (A549)-transplanted mice with per os administration. Thus, **8g** (named as **DDO-6318**) was identified as a new and orally-active natural-product-like caged xanthone, and also a promising antitumor agent for further clinical development.

## Figures and Tables

**Table 1:**
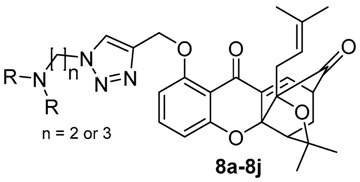
In vitro antiproliferative activity of the 1,2,3-triazole-bearing caged xanthone compounds.

Cpd	-NRR	n	*IC*_50_ (μM)					
			A549	A549/Taxol	A549/Cisplatin	HepG2	HCT116	U2OS
**8a**	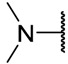	2	1.95 ± 0.40	1.22 ± 0.21	1.48 ± 0.10	2.12 ± 0.41	1.64 ± 0.26	2.22 ± 0.09
**8b**	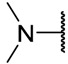	3	2.62 ± 0.20	1.30 ± 0.08	1.66 ± 0.11	1.36 ± 0.16	2.27 ± 0.05	2.73 ± 0.05
**8c**	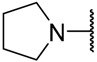	2	2.58 ± 0.39	1.29 ± 0.07	1.34 ± 0.09	1.19 ± 0.31	0.98 ± 0.31	2.01 ± 0.20
**8d**	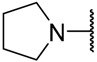	3	2.62 ± 0.04	1.70 ± 0.12	0.89 ± 0.24	4.48 ± 0.29	1.02 ± 0.03	3.05 ± 0.07
**8e**	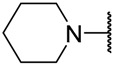	2	2.17 ± 0.12	2.03 ± 0.11	1.29 ± 0.48	4.85 ± 0.39	2.14 ± 0.18	4.66 ± 0.03
**8f**	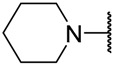	3	1.07 ± 0.28	1.29 ± 0.08	1.32 ± 0.12	6.30 ± 0.48	1.39 ± 0.04	2.07 ± 0.11
**8g**	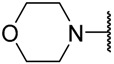	2	0.31 ± 0.02	0.42 ± 0.05	0.33 ± 0.07	3.79 ± 0.43	0.28 ± 0.03	0.95 ± 0.21
**8h**	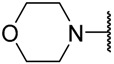	3	1.03 ± 0.06	0.55 ± 0.08	0.36 ± 0.04	3.07 ± 0.13	0.95 ± 0.12	0.73 ± 0.07
**8i**	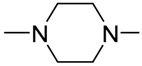	2	3.33 ± 0.49	2.12 ± 0.09	0.78 ± 0.08	5.35 ± 0.23	6.15 ± 0.86	9.51 ± 1.52
**8j**	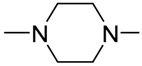	3	0.93 ± 0.08	0.82 ± 0.08	1.29 ± 0.11	2.91 ± 0.03	1.09 ± 0.21	5.86 ± 0.21
**DDO-6101**	—		2.59 ± 0.17	0.46 ± 0.03	2.11 ± 0.10	2.04 ± 0.08	0.71 ± 0.10	5.35 ± 0.08
**GA**	—		2.02 ± 0.02	0.29 ± 0.04	2.50 ± 0.06	2.08 ± 0.07	0.34 ± 0.08	4.67 ± 0.11

**Table 2:** Druglike properties of the caged xanthone derivatives **8a**–**8j**.

Cpd	PSA_2D (Å2) *^a^*	Log D 7.4 *^b^*	Absorption_Level *^c^*	S (mM) *^d^*
8a	92.614	2.1	0	1.12
8b	92.614	1.8	0	1.54
8c	92.614	2.2	0	1.40
8d	92.614	1.6	0	1.92
8e	92.614	2.9	0	0.56
8f	92.614	2.7	1	0.88
8g	101.544	1.3	0	3.02
8h	101.544	0.9	0	3.70
8i	95.967	1.0	0	3.22
8j	95.967	0.7	0	4.02
DDO-6101	73.277	2.9	0	<0.005
GA	120.323	3.6	1	<0.005

*^a^* Calculated value with Accelrys Discovery Studio 4.0; *^b^* Measured value of log D 7.4 on a Gemini Profiler instrument (pION); *^c^* The data were predicted with Accelrys Discovery Studio 4.0. Absorption_level (0 = good, 1 = moderate); *^d^* The solubility in water.

**Table 3:** The inhibitory effect of **8g** with intravenous administration on the growth of A549 transplanted mice.

Group	Dose (mg/kg)	Body Weight (g)		Weight of Tumor (g)	Inhibitory Rate (%)
		Pre-Dose	After-Dose		
control	0	20.95 ± 1.22	29.25 ± 3.37	1.36 ± 0.20	0.0
**8g**	5	20.80 ± 1.53	28.53 ± 2.81	0.94 ± 0.24 *^a^*	30.88
**8g**	10	21.40 ± 1.81	28.08 ± 2.66	0.65 ± 0.17 *^b^*	52.21
**8g**	20	20.67 ± 1.30	27.73 ± 2.78	0.40 ± 0.14 *^b^*	71.32
**DDO-6101**	20	21.13 ± 1.22	27.69 ± 1.90	0.89 ± 0.18 *^b^*	34.56
**5-FU**	20	20.25 ± 1.27	24.43 ± 2.62	0.48 ± 0.17 *^b^*	64.71

*^a^* *p* < 0.05; *^b^* *p* < 0.01 vs. control.

**Table 4:** The inhibitory effect of **8g** with per os administration on the growth of A549-transplanted mice.

Group	Dose (mg/kg)	Body Weight (g)		Weight of Tumor (g)	Inhibitory Rate (%)
		Pre-Dose	After-Dose		
control	0	20.86 ± 1.18	28.55 ± 2.97	1.40 ± 0.22	0.0
**8g**	50	20.67 ± 1.20	27.73 ± 2.78	0.47 ± 0.12 *^a^*	66.43
**DDO-6101**	50	21.13 ± 1.02	26.69 ± 1.90	1.10 ± 0.25 *^b^*	21.43

*^a^* *p* < 0.05; *^b^* *p* < 0.01 vs. control.

## Supplementary Materials

Supplementary Materials are available online. **1**: ^1^H-NMR, ^13^C-NMR, HRMS spectra for target compounds; Table S1: Data for Figure 3.
