# Supplementary material for: ‘Click Chemistry’ Synthesis of Novel Natural Product-Like Caged Xanthones Bearing a 1,2,3-Triazole Moiety with Improved Druglike Properties as Orally Active Antitumor Agents
Source: Molecules. 2017 Oct 27;22(11):1834. doi: 10.3390/molecules22111834 (PMC6150271; doi:10.3390/molecules22111834)
Supplement: Supplementary file 1 [file molecules-22-01834-s001.pdf]

Supplementary Materials

'Click Chemistry' Synthesis of Novel Natural Product-Like Caged Xanthones Bearing a 1,2,3-Triazole Moiety with Improved Druglike Properties as Orally Active Antitumor Agents

Xiang Li [1,2], Yue Wu [1,3], Yanyan Wang [1,3], Qidong You [1,3,*] and Xiaojin Zhang [1,4,*]

[1] *Jiangsu Key Laboratory of Drug Design and Optimization, State Key Laboratory of Natural Medicines, China Pharmaceutical University, Nanjing 210009, China*
[2] *Department of Pharmaceutical Engineering, China Pharmaceutical University, Nanjing 211198, China*
[3] *Department of Medicinal Chemistry, China Pharmaceutical University, Nanjing 21009, China*
[4] *Department of Organic Chemistry, China Pharmaceutical University, Nanjing 211198, China*

*Corresponding author
E-mail addresses: youqd@163.com (Q. You)
E-mail addresses: zxj@cpu.edu.cn (X. Zhang)

Table of Contents:

1. ^{1}H NMR, ^{13}C NMR, HRMS spectra for target compounds……………. S3-S15
2. TableS1. Data for Figure 3…………………………………………… S15

1. Spectra for target compounds

^{1}H NMR(300 MHz) of **8a** in $CDCl_3$

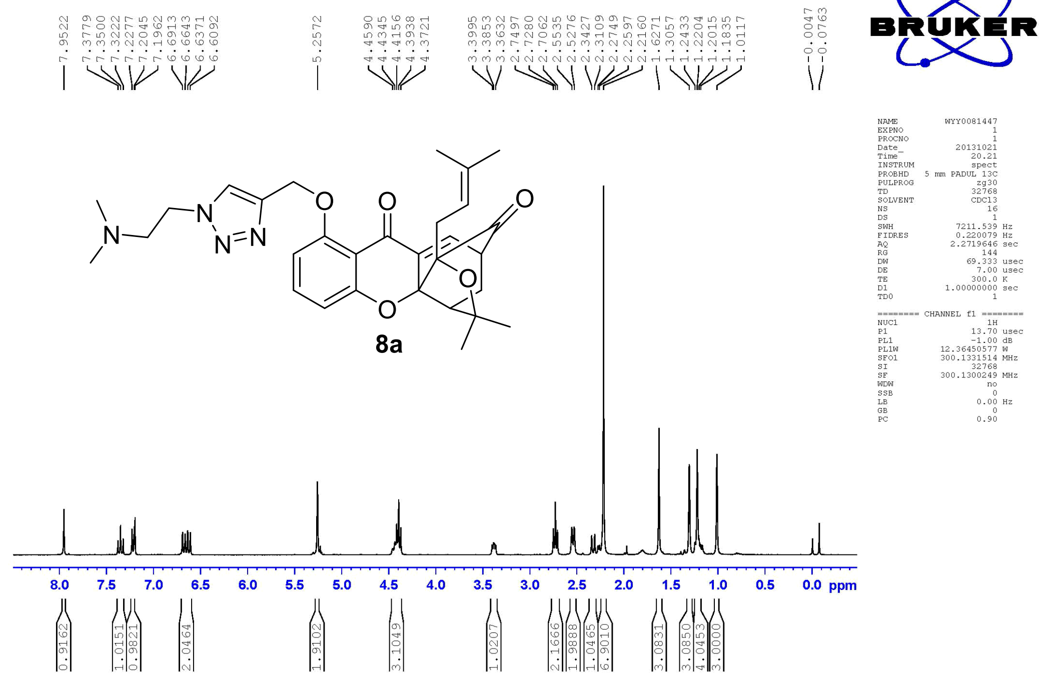

^{13}C NMR(75 MHz) of **8a** in $CDCl_3$

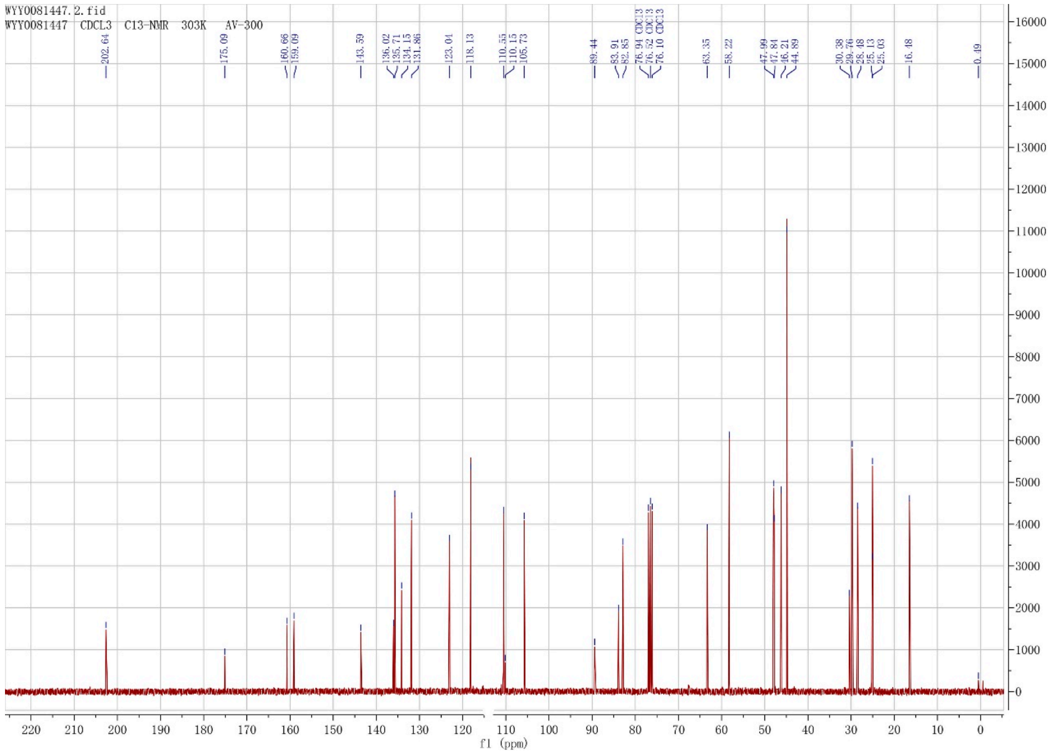

HRMS of **8a**

**Sample Name**		**Position**	P2-B3	**Instrument Name**	Instrument 1	**User Name**	
**Inj Vol**	0.5	**InjPosition**		**SampleType**	Sample	**IRM Calibration Status**	Success
**Data Filename**	WYY0081447-p.d	**ACQ Method**		**Comment**		**Acquired Time**	10/30/2013 6:42:48 PM

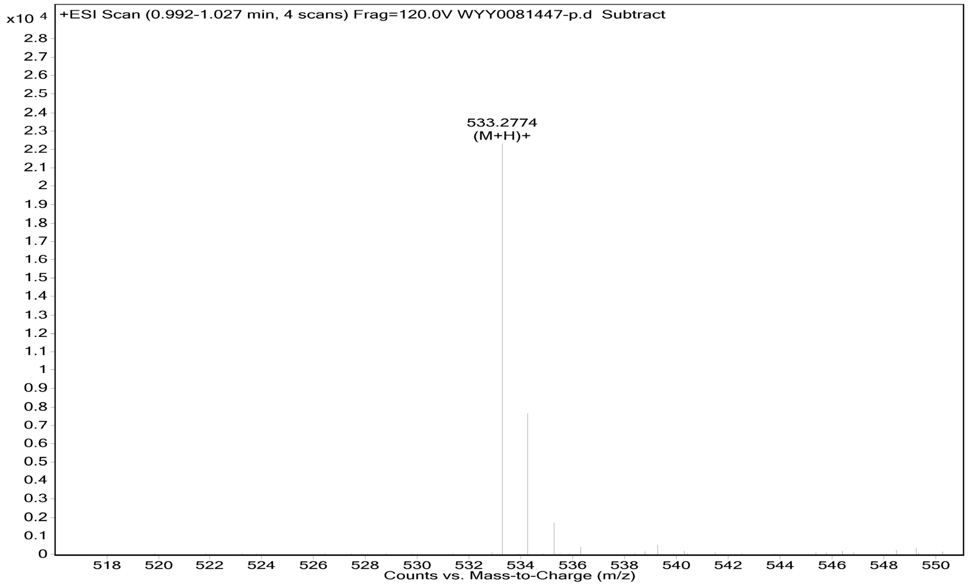

^{1}H NMR(300 MHz) of **8b** in $CDCl_3$

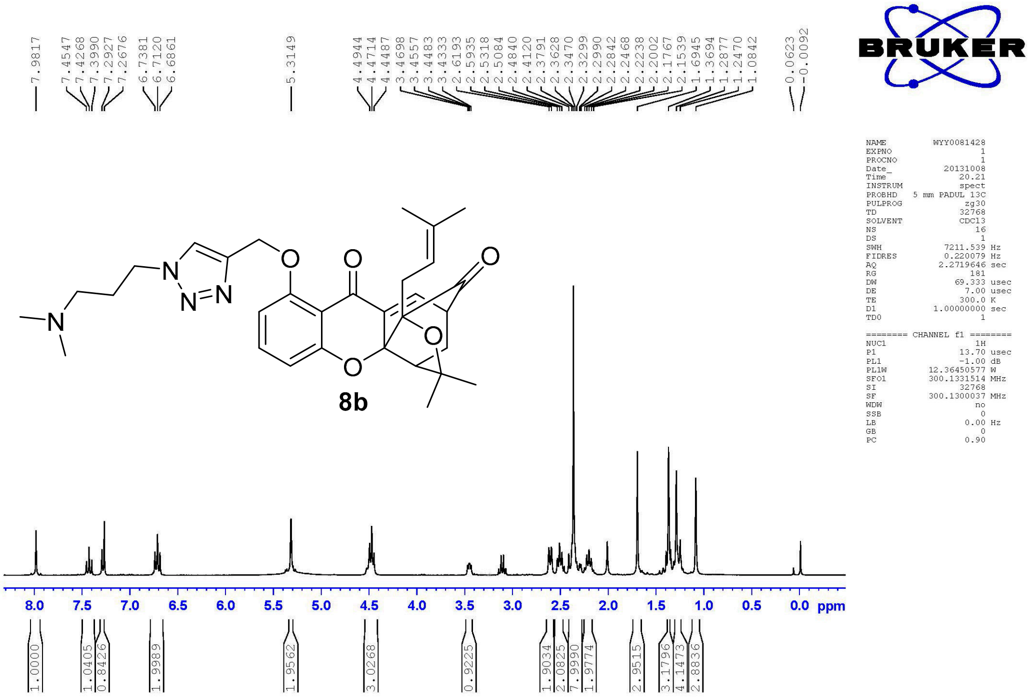

HRMS of **8b**

**Sample Name**		**Position**	P2-D3	**Instrument Name**	Instrument 1	**User Name**	
**Inj Vol**	0.5	**InjPosition**		**SampleType**	Sample	**IRM Calibration Status**	Success
**Data Filename**	WYY0081428-p.d	**ACQ Method**		**Comment**		**Acquired Time**	10/12/2013 7:51:47 PM

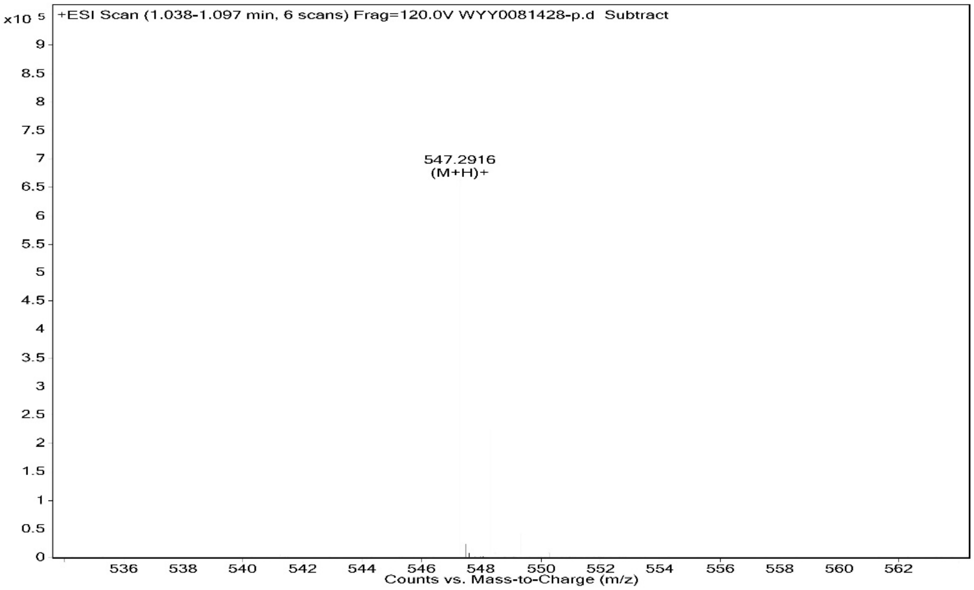

^{1}H NMR(300 MHz) of **8c** in $CDCl_3$

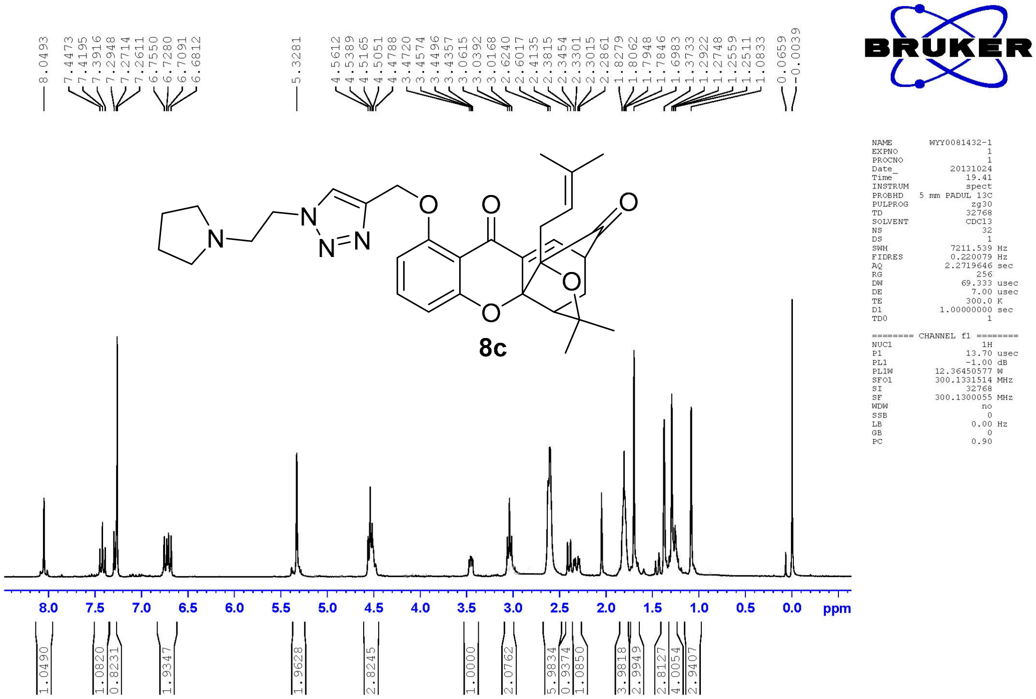

^{13}C NMR(75 MHz) of **8c** in $CDCl_3$

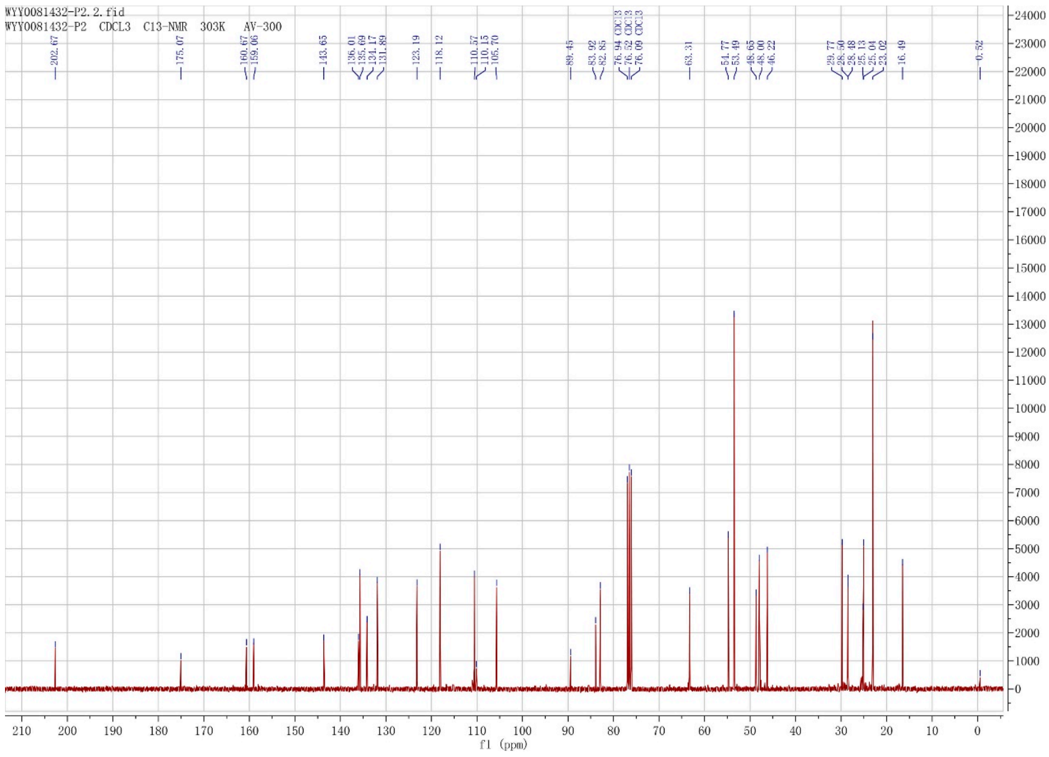

HRMS of **8c**

**Sample Name**		**Position**	P2-D4	**Instrument Name**	Instrument 1	**User Name**	
**Inj Vol**	0.5	**InjPosition**		**SampleType**	Sample	**IRM Calibration Status**	Success
**Data Filename**	WYY0081432-p.d	**ACQ Method**		**Comment**		**Acquired Time**	10/12/2013 7:54:49 PM

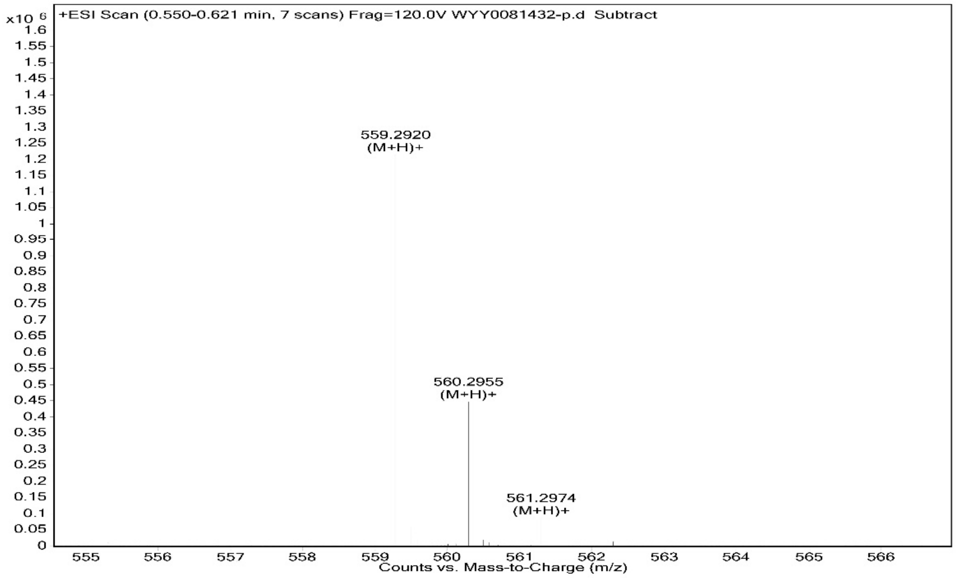

^{1}H NMR(300 MHz) of **8d** in $CDCl_3$

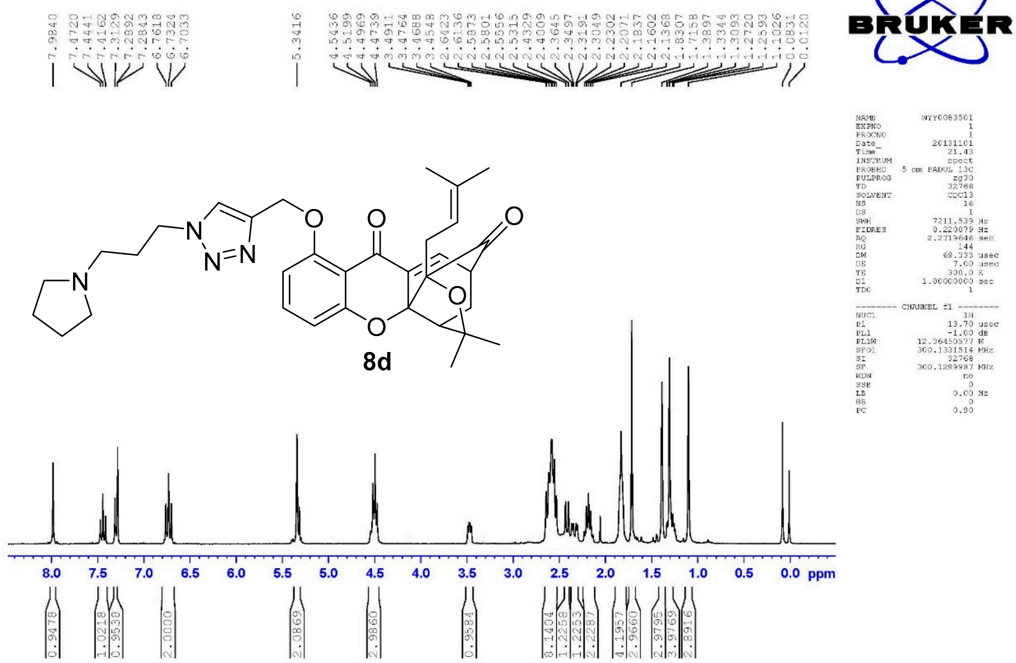

HRMS of **8d**

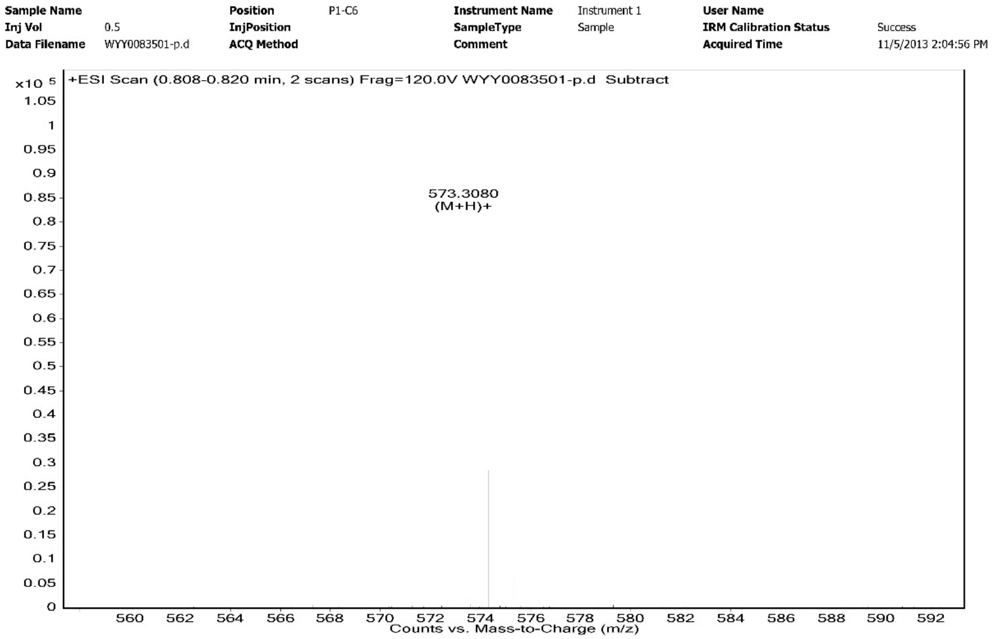

^{1}H NMR(300 MHz) of **8e** in $CDCl_3$

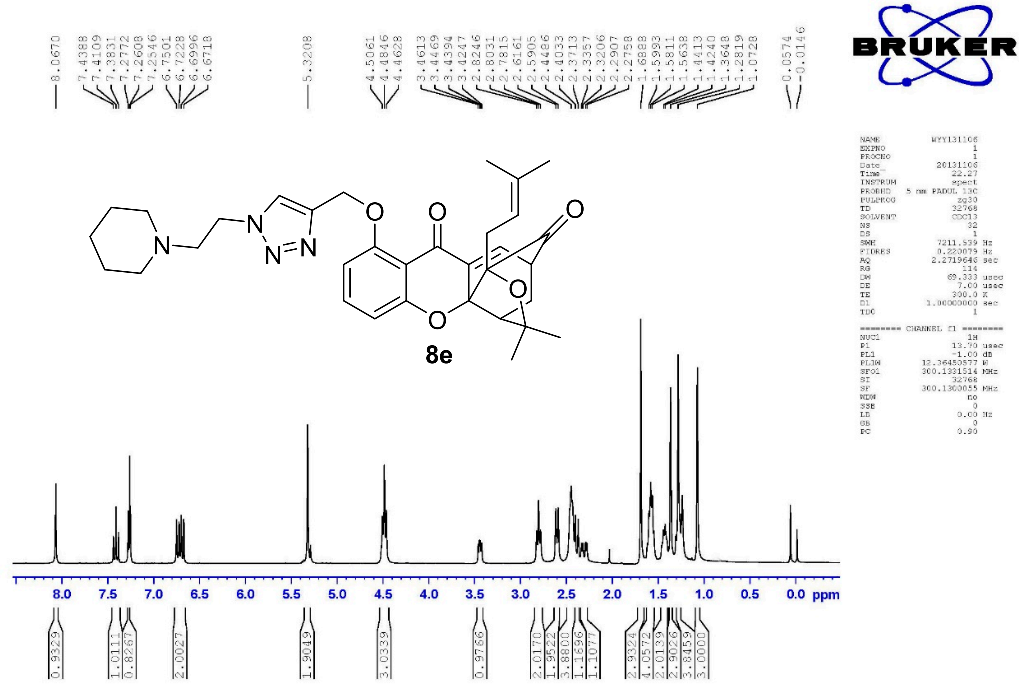

^{13}C NMR(75 MHz) of **8e** in $CDCl_3$

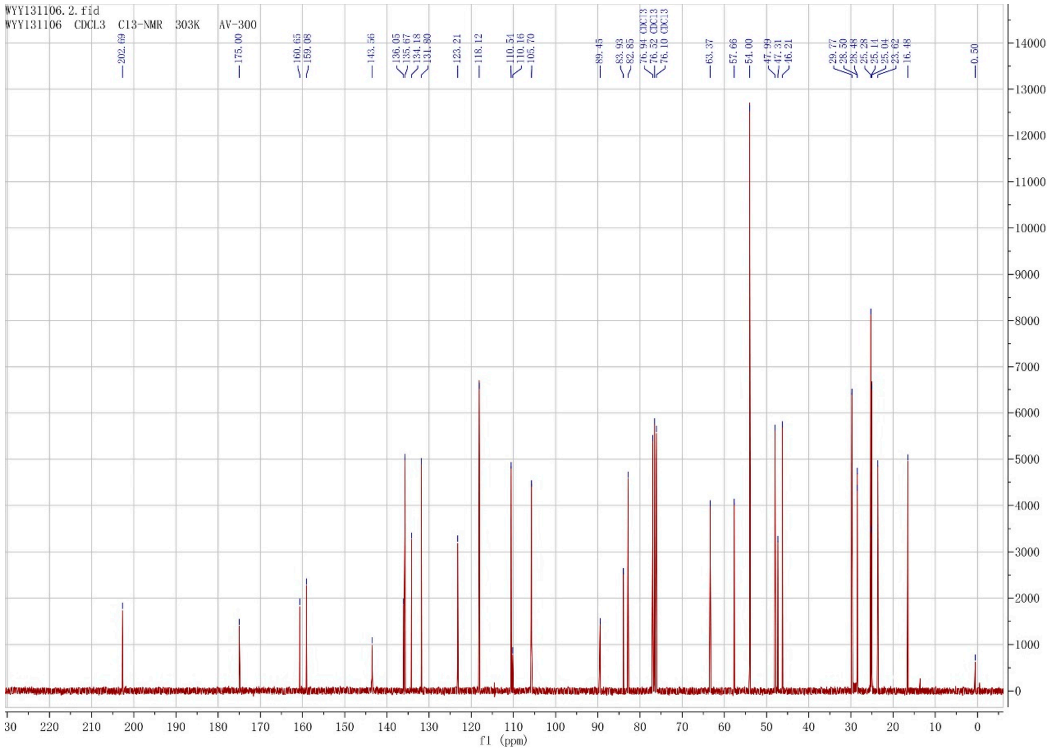

HRMS of **8e**

**Sample Name**		**Position**	P2-B1	**Instrument Name**	Instrument 1	**User Name**	
**Inj Vol**	0.5	**InjPosition**		**SampleType**	Sample	**IRM Calibration Status**	Success
**Data Filename**	WYY0083505-p.d	**ACQ Method**		**Comment**		**Acquired Time**	11/13/2013 2:46:29 PM

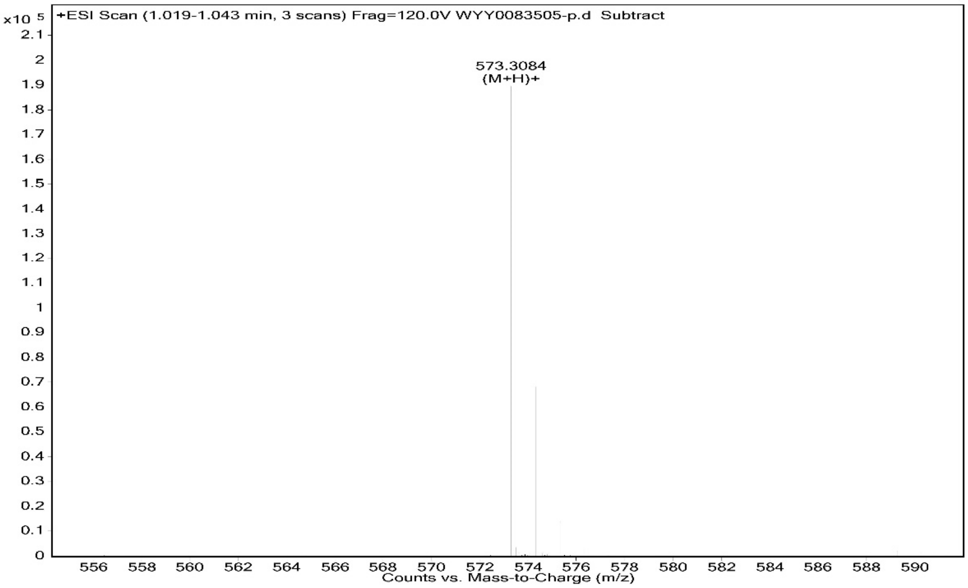

^{1}H NMR(300 MHz) of **8f** in $CDCl_3$

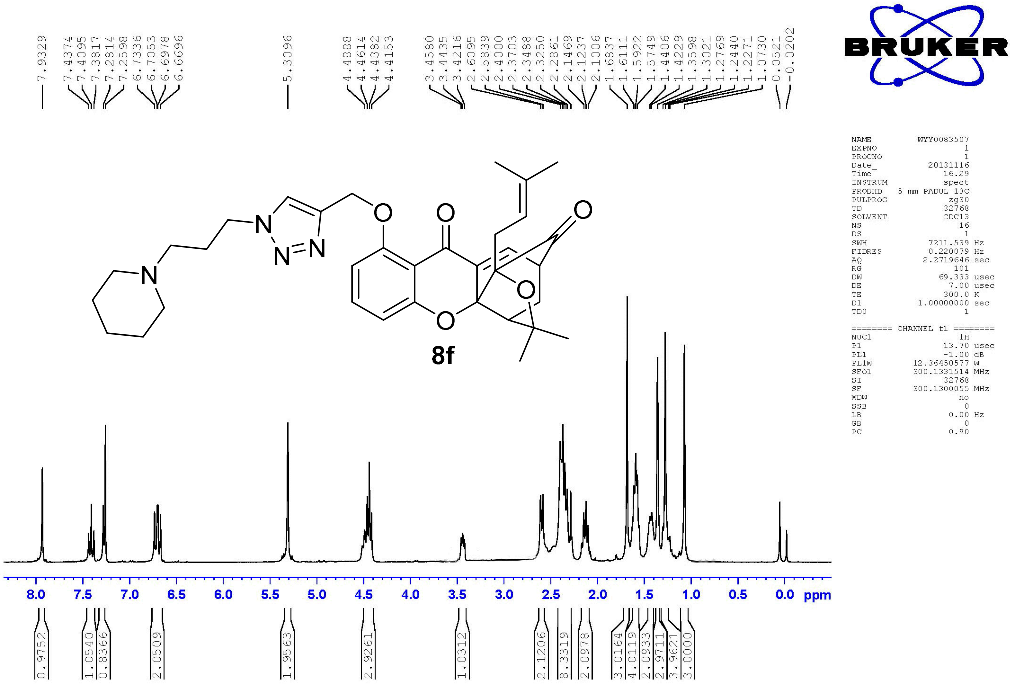

HRMS of **8f**

Sample Name		Position	P1-D8	Instrument Name	Instrument 1	User Name	
Inj Vol	0.5	InjPosition		SampleType	Sample	IRM Calibration Status	Success
Data Filename	WYY0083507-p.d	ACQ Method		Comment		Acquired Time	11/19/2013 4:17:43 PM

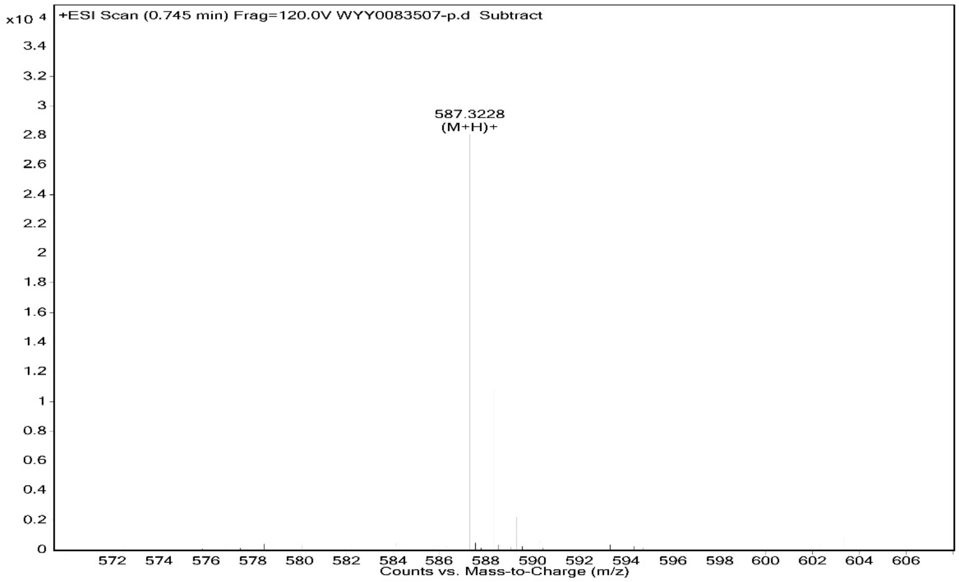

^{1}H NMR(300 MHz) of **8g** in $CDCl_3$

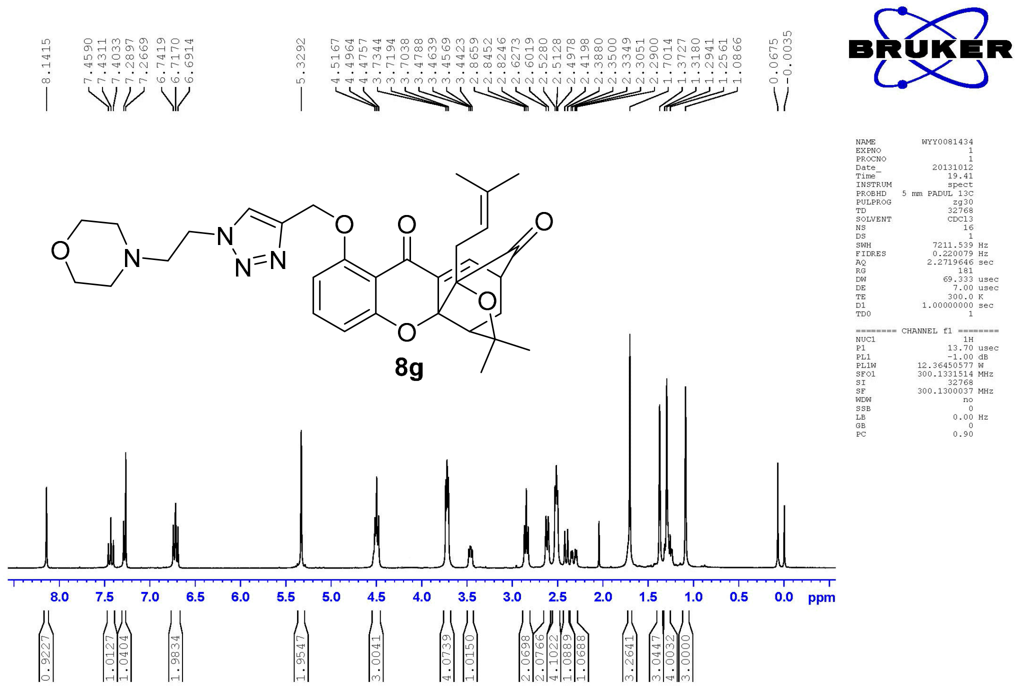

^{13}C NMR(75 MHz) of **8g** in $CDCl_3$

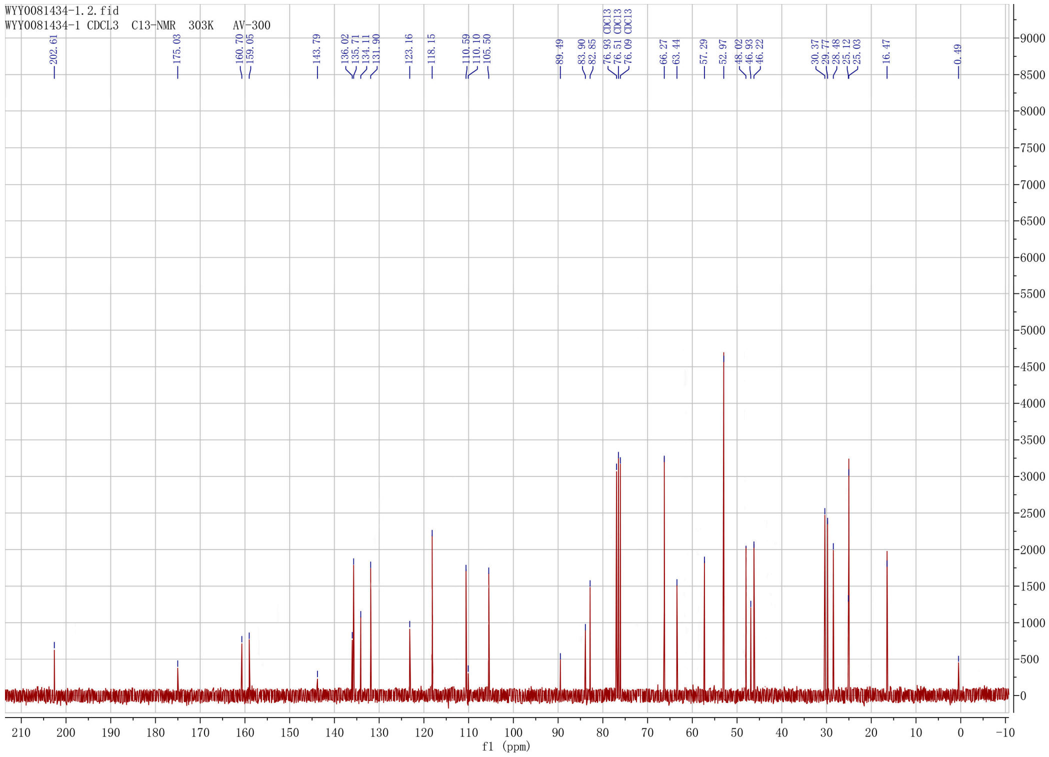

HRMS of **8g**

**Sample Name**		**Position**	P2-E8	**Instrument Name**	Instrument 1	**User Name**	
**Inj Vol**	0.5	**InjPosition**		**SampleType**	Sample	**IRM Calibration Status**	Success
**Data Filename**	WYY0081434-p0002.d	**ACQ Method**		**Comment**		**Acquired Time**	10/13/2013 6:00:40 PM

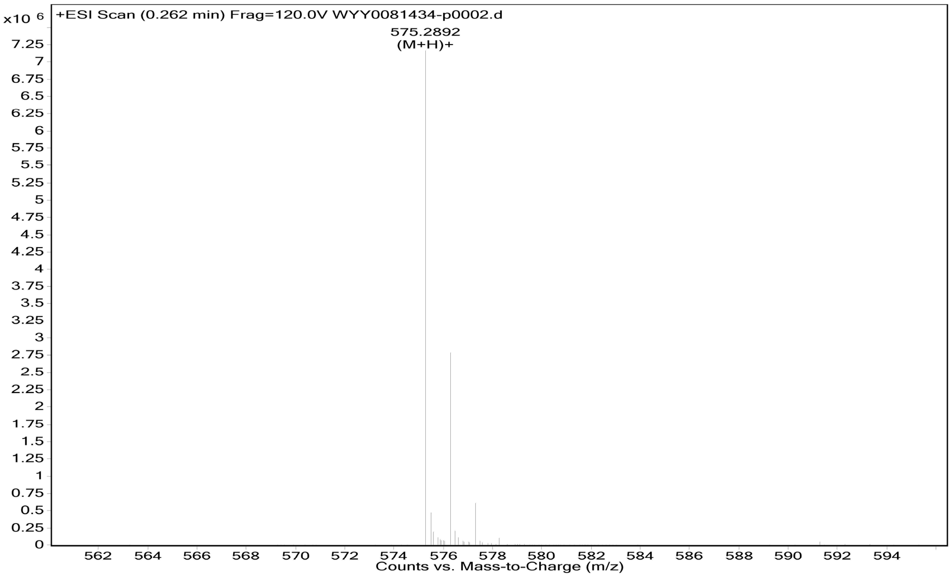

^{1}H NMR(300 MHz) of **8h** in $CDCl_3$

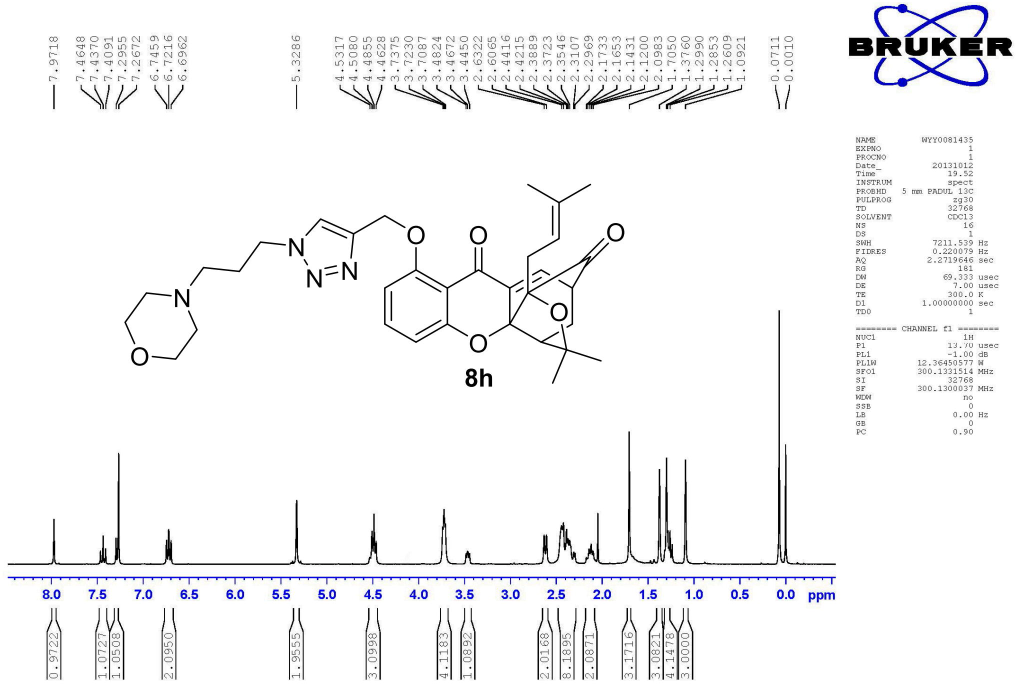

^{13}C NMR(75 MHz) of **8h** in $CDCl_3$

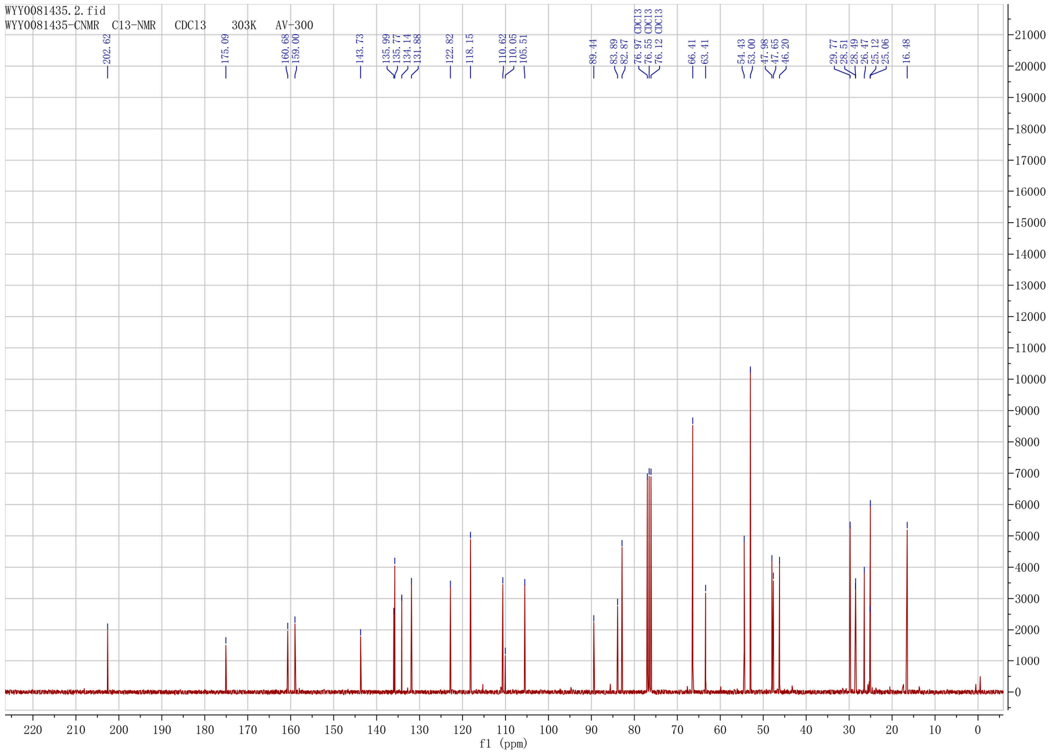

HRMS of **8h**

Sample Name		Position	P2-E9	Instrument Name	Instrument 1	User Name	
Inj Vol	2	InjPosition		SampleType	Sample	IRM Calibration Status	Success
Data Filename	WYY0081435-p0003.d	ACQ Method		Comment		Acquired Time	10/13/2013 6:03:48 PM

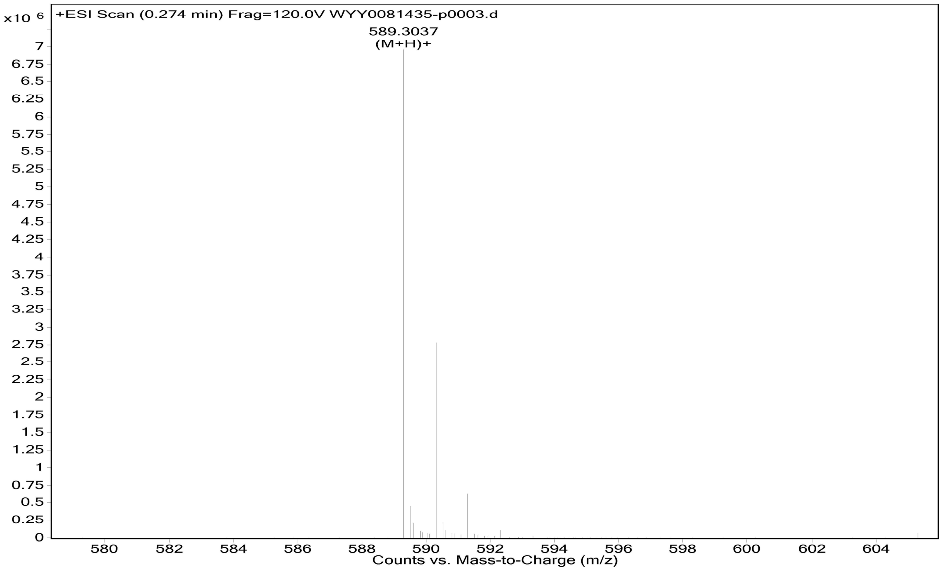

^{1}H NMR(300 MHz) of **8i** in $CDCl_3$

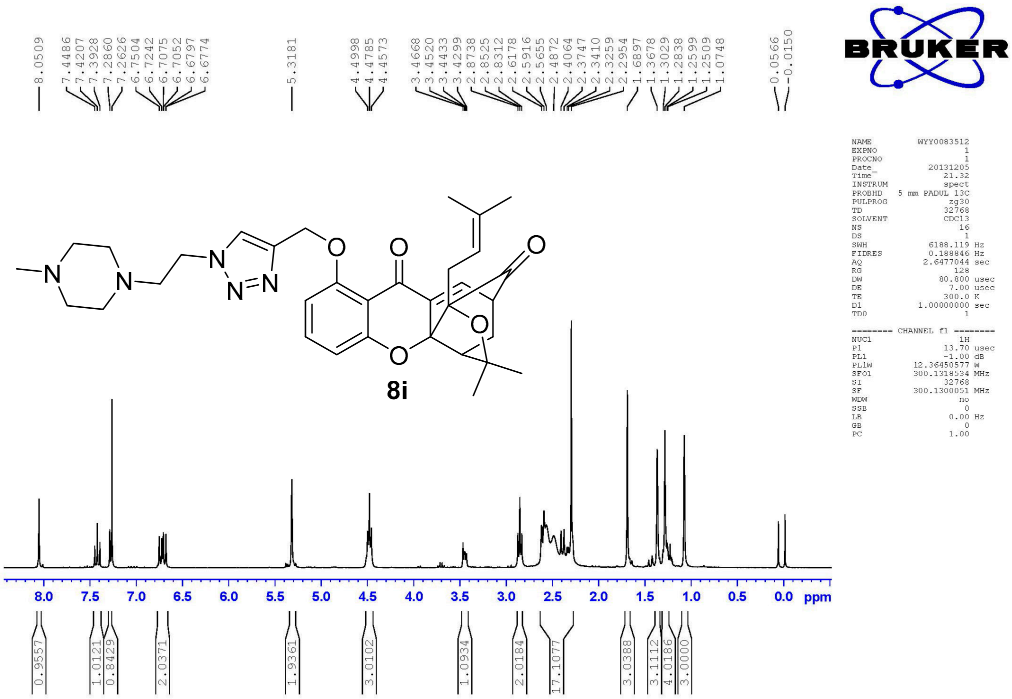

HRMS of **8i**

**Sample Name**		**Position**	P1-C9	**Instrument Name**	Instrument 1	**User Name**	
**Inj Vol**	0.6	**InjPosition**		**SampleType**	Sample	**IRM Calibration Status**	Success
**Data Filename**	JL-X5-p.d	**ACQ Method**		**Comment**		**Acquired Time**	12/12/2013 12:59:57 PM

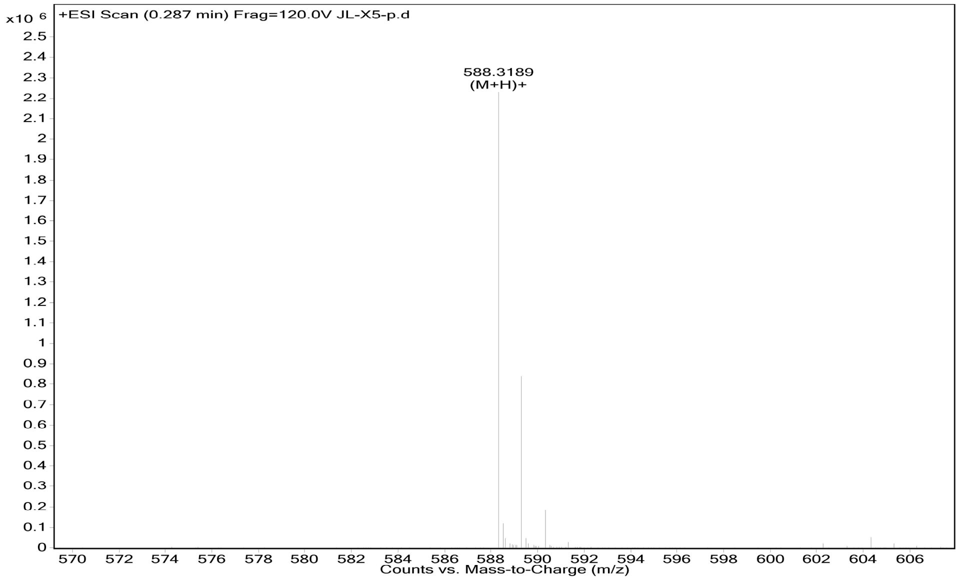

^{1}H NMR(300 MHz) of **8j** in $CDCl_3$

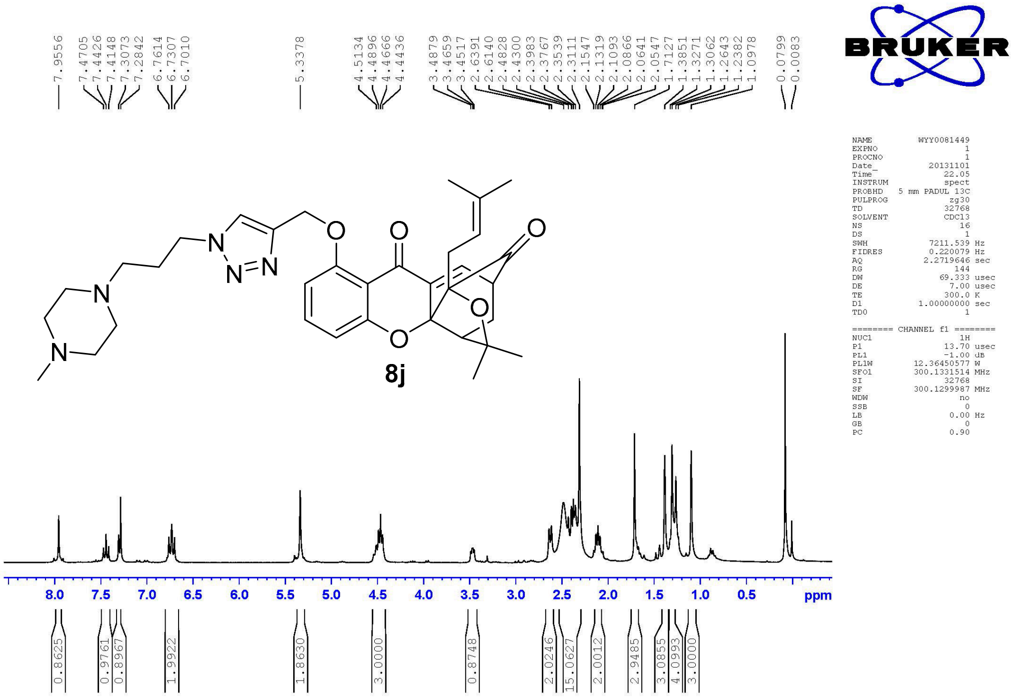

HRMS of **8j**

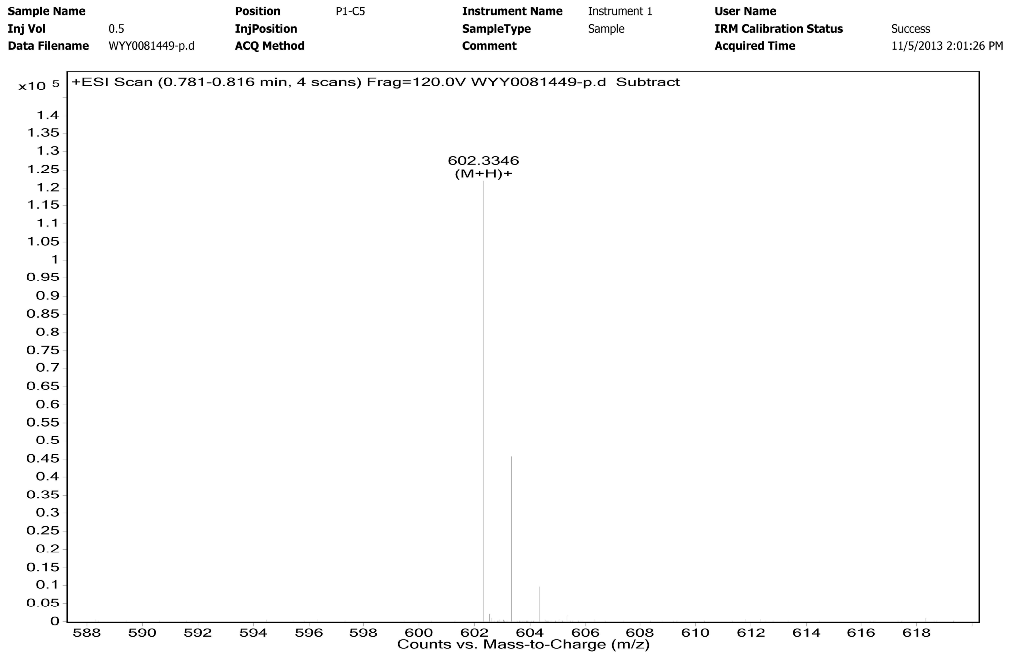

2. Data for Figure 3.

Table S1. Data of membrane permeability for Figure 3

Cpd	P_e (10^{-6} cm/s) [a]		
	pH=5.0	pH=6.2	pH=7.4
**8a**	27.9±3.3	58.8±2.1	91.4±9.8
**8b**	16.3±1.2	41.7±3.2	76.1±6.4
**8c**	42.3±1.5	75.4±6.9	91.9±6.0
**8d**	15.4±2.1	52.8±1.6	66.4±8.4
**8e**	47.8±4.7	57.2±1.4	78.2±8.8
**8f**	34.9±3.6	55.1±0.8	68.7±5.3
**8g**	51.3±4.8	63.1±5.6	74.4±6.8
**8h**	40.0±0.1	58.0±6.1	72.6±0.1
**8i**	11.3±0.6	41.5±1.3	67.5±4.2
**8j**	9.7±0.5	43.2±0.7	64.9±5.4
**DDO-6101**	23.6±5.5	25.8±1.1	18.0±3.9
**GA**	0.9±0.5	1.8±0.2	2.3±0.3
Ketoprofen [a]	—	—	1.8±0.3
Propranolol [a]	—	—	127.5±15.8

[a] Ketoprofen and propranolol are internal standards in permeability determinations. Data are expressed as the mean P_e from three independent experiments.
